# Molecular Architecture of Early Dissemination and Massive Second Wave of the SARS-CoV-2 Virus in a Major Metropolitan Area

**DOI:** 10.1128/mBio.02707-20

**Published:** 2020-10-30

**Authors:** S. Wesley Long, Randall J. Olsen, Paul A. Christensen, David W. Bernard, James J. Davis, Maulik Shukla, Marcus Nguyen, Matthew Ojeda Saavedra, Prasanti Yerramilli, Layne Pruitt, Sishir Subedi, Hung-Che Kuo, Heather Hendrickson, Ghazaleh Eskandari, Hoang A. T. Nguyen, J. Hunter Long, Muthiah Kumaraswami, Jule Goike, Daniel Boutz, Jimmy Gollihar, Jason S. McLellan, Chia-Wei Chou, Kamyab Javanmardi, Ilya J. Finkelstein, James M. Musser

**Affiliations:** a Center for Molecular and Translational Human Infectious Diseases Research, Department of Pathology and Genomic Medicine, Houston Methodist Research Institute, Houston Methodist Hospital, Houston, Texas, USA; b Department of Pathology and Laboratory Medicine, Weill Cornell Medical College, New York, New York, USA; c Department of Microbiology and Immunology, Weill Cornell Medical College, New York, New York, USA; d Consortium for Advanced Science and Engineering, University of Chicago, Chicago, Illinois, USA; e Computing, Environment and Life Sciences, Argonne National Laboratory, Lemont, Illinois, USA; f Department of Molecular Biosciences, The University of Texas at Austin, Austin, Texas, USA; g Institute for Cell and Molecular Biology, The University of Texas at Austin, Austin, Texas, USA; h CCDC Army Research Laboratory-South, University of Texas, Austin, Texas, USA; i Center for Systems and Synthetic Biology, University of Texas at Austin, Austin, Texas, USA; Louis Stokes Veterans Affairs Medical Center

**Keywords:** SARS-CoV-2, COVID-19 disease, genome sequencing, molecular population genomics, evolution, COVID-19

## Abstract

We sequenced the genomes of 5,085 severe acute respiratory syndrome coronavirus 2 (SARS-CoV-2) strains causing two coronavirus disease 2019 (COVID-19) disease waves in metropolitan Houston, TX, an ethnically diverse region with 7 million residents. The genomes were from viruses recovered in the earliest recognized phase of the pandemic in Houston and from viruses recovered in an ongoing massive second wave of infections. The virus was originally introduced into Houston many times independently. Virtually all strains in the second wave have a Gly614 amino acid replacement in the spike protein, a polymorphism that has been linked to increased transmission and infectivity. Patients infected with the Gly614 variant strains had significantly higher virus loads in the nasopharynx on initial diagnosis. We found little evidence of a significant relationship between virus genotype and altered virulence, stressing the linkage between disease severity, underlying medical conditions, and host genetics. Some regions of the spike protein—the primary target of global vaccine efforts—are replete with amino acid replacements, perhaps indicating the action of selection. We exploited the genomic data to generate defined single amino acid replacements in the receptor binding domain of spike protein that, importantly, produced decreased recognition by the neutralizing monoclonal antibody CR3022. Our report represents the first analysis of the molecular architecture of SARS-CoV-2 in two infection waves in a major metropolitan region. The findings will help us to understand the origin, composition, and trajectory of future infection waves and the potential effect of the host immune response and therapeutic maneuvers on SARS-CoV-2 evolution.

## Abstract (precis)

There is concern about second and subsequent waves of COVID-19 caused by the SARS-CoV-2 coronavirus occurring in communities globally that had an initial disease wave. Metropolitan Houston, TX, with a population of 7 million, is experiencing a massive second disease wave that began in late May 2020. To understand SARS-CoV-2 molecular population genomic architecture and evolution and the relationship between virus genotypes and patient features, we sequenced the genomes of 5,085 SARS-CoV-2 strains from these two waves. Our report provides the first molecular characterization of SARS-CoV-2 strains causing two distinct COVID-19 disease waves.

## INTRODUCTION

Pandemic disease caused by the severe acute respiratory syndrome coronavirus 2 (SARS-CoV-2) virus is now responsible for massive human morbidity and mortality worldwide (1–5). The virus was first documented to cause severe respiratory infections in Wuhan, China, beginning in late December 2019 (6–9). Global dissemination occurred extremely rapidly and has affected major population centers on most continents (10, 11). In the United States, the Seattle and the New York City (NYC) regions have been especially important centers of coronavirus disease 2019 (COVID-19) caused by SARS-CoV-2. For example, as of 19 August 2020, there were 227,419 confirmed SARS-CoV-2 cases in NYC, causing 56,831 hospitalizations and 19,005 confirmed fatalities and 4,638 probable fatalities (12). Similarly, in Seattle and King County, WA, 17,989 SARS-CoV-2-positive patients and 696 deaths had been reported as of 18 August 2020 (13).

**FIG 1:**
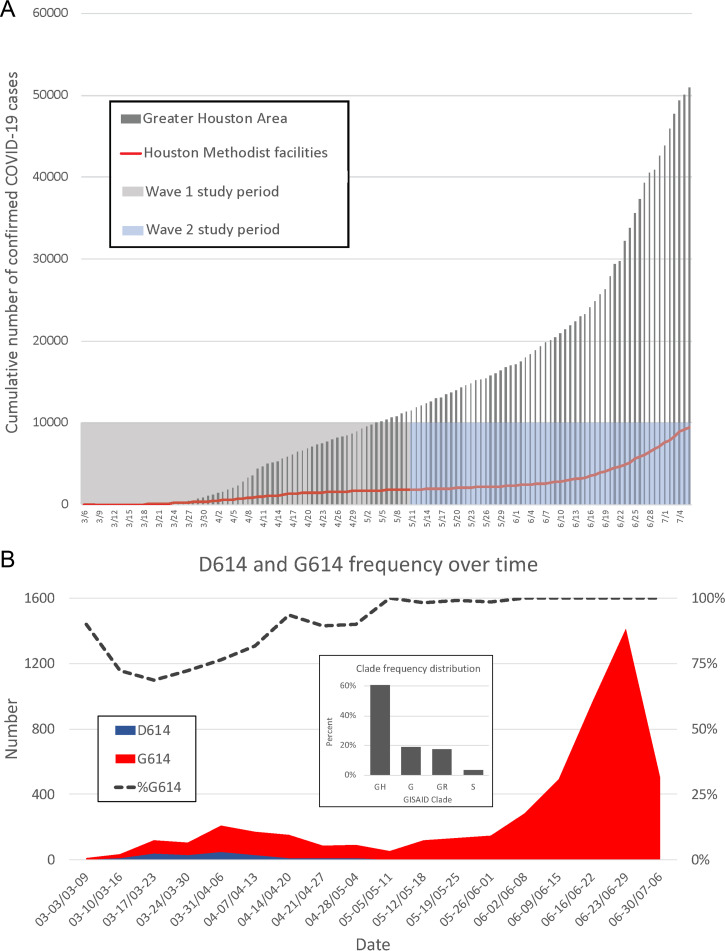
(A) Confirmed COVID-19 cases in the Greater Houston Metropolitan region. Data represent cumulative number of COVID-19 patients over time through 7 July 2020. Counties include Austin, Brazoria, Chambers, Fort Bend, Galveston, Harris, Liberty, Montgomery, and Waller. The shaded area represents the time period (indicated as month/day along the *x* axis) during which virus genomes characterized in this study were recovered from COVID-19 patients. The red line represents the number of COVID-19 patients diagnosed in the Houston Methodist Hospital Molecular Diagnostic Laboratory. (B) Distribution of strains with either the Asp614 or Gly614 amino acid variant in spike protein among the two waves of COVID-19 patients diagnosed in the Houston Methodist Hospital Molecular Diagnostic Laboratory. The large inset shows major clade frequency for the time frame studied (indicated as month-day to month-day along the *x* axis).

The Houston metropolitan area is the fourth largest and most ethnically diverse city in the United States, with a population of approximately 7 million (14). The 2,400-bed Houston Methodist Hospital health system has seven hospitals and serves a large, multiethnic, and socioeconomically diverse patient population throughout greater Houston (13, 14). The first COVID-19 case in metropolitan Houston was reported on 5 March 2020, with community spread occurring 1 week later (15). Many of the first cases in our region were associated with national or international travel in areas known to have SARS-CoV-2 virus outbreaks (15). A central molecular diagnostic laboratory serving all Houston Methodist hospitals and our very early adoption of a molecular test for the SARS-CoV-2 virus permitted us to rapidly identify SARS-CoV-2-positive patients and interrogate genomic variation among strains causing early infections in the greater Houston area. Our analysis of SARS-CoV-2 genomes causing disease in Houston has continued unabated since early March and is ongoing. Genome sequencing and related efforts were expanded extensively in late May as we recognized that a prominent second wave was under way (Fig. 1).

Here, we report that SARS-CoV-2 was introduced to the Houston area many times, independently, from diverse geographic regions, with virus genotypes representing genetic clades causing disease in Europe, Asia, and South America and elsewhere in the United States. There was widespread community dissemination soon after COVID-19 cases were reported in Houston. Detection of strains with a Gly614 amino acid replacement in the spike protein, a polymorphism that has been linked to increased transmission and *in vitro* cell infectivity, increased significantly over time and caused virtually all COVID-19 cases in the massive second disease wave. Patients infected with strains with the Gly614 variant had significantly higher virus loads in the nasopharynx on initial diagnosis. Some naturally occurring single amino acid replacements in the receptor binding domain (RBD) of spike protein resulted in decreased reactivity with a neutralizing monoclonal antibody, consistent with the idea that some virus variants arise due to host immune pressure.

## RESULTS

### Description of metropolitan Houston.

Houston, TX, is located in the southwestern United States, 50 miles inland from the Gulf of Mexico. It is the most ethnically diverse city in the United States (14). Metropolitan Houston is comprised predominantly of Harris County plus parts of eight contiguous surrounding counties. In the aggregate, the metropolitan area includes 9,444 square miles. The estimated population size of metropolitan Houston is 7 million (https://www.houston.org/houston-data).

### Epidemic curve characteristics over two disease waves.

The first confirmed case of COVID-19 in the Houston metropolitan region was reported on 5 March 2020 (15), and the first confirmed case diagnosed in Houston Methodist hospitals was reported on 6 March 2020. The epidemic curve indicated a first wave of COVID-19 cases that peaked around 11 to 15 April, followed by a decline in cases until 11 May. Soon thereafter, the slope of the case curve increased, with a very sharp uptick in confirmed cases beginning on 12 June (Fig. 1B). We consider 11 May to represent the transition between waves, as this date represents the inflection point of the curve of cumulative new cases and had the absolute lowest number of new cases in the mid-May time period. Thus, for the data presented here, wave 1 is defined as 5 March through 11 May 2020, and wave 2 is defined as 12 May through 7 July 2020. Epidemiologic trends within the Houston Methodist Hospital population were mirrored by data from Harris County and the greater metropolitan Houston region (Fig. 1A). Through 7 July, 25,366 COVID-19 cases were reported in Houston, 37,776 cases in Harris County, and 53,330 in metropolitan Houston, including 9,823 cases in Houston Methodist Hospital facilities (inpatients and outpatients) (https://www.tmc.edu/coronavirus-updates/infection-rate-in-the-greater-houston-area/ and https://harriscounty.maps.arcgis.com/apps/opsdashboard/index.html#/c0de71f8ea484b85bb5efcb7c07c6914).

During the first wave (early March through May 11), 11,476 COVID-19 cases were reported in Houston, including 1,729 cases in the Houston Methodist Hospital system. Early in the first wave (from March 5 through 30 March 2020), we tested 3,080 patient specimens. Of these, 406 (13.2%) samples were positive for SARS-CoV-2, representing 40% (358/898) of all confirmed cases in metropolitan Houston during that time period. As our laboratory was the first hospital-based facility to have the capacity for molecular testing for SARS-CoV-2 on site, our strain samples are likely representative of COVID-19 infections during the first wave.

For the entire study period (5 March through 7 July 2020), we tested 68,418 specimens from 55,800 patients. Of these, 9,121 patients (16.4%) had a positive test result, representing 17.1% (9,121/53,300) of all confirmed cases in metropolitan Houston. Thus, our strain samples are also representative of those responsible for COVID-19 infections in the massive second wave.

To test the hypothesis that, on average, the two waves affected different groups of patients, we analyzed individual patient characteristics (hospitalized and nonhospitalized) in each wave. Consistent with this hypothesis, we found significant differences in the COVID-19 patients in each wave (see Table S1 in the supplemental material). For example, patients in the second wave were significantly younger, had fewer comorbidities, were more likely to be Hispanic/Latino (by self-report), and lived in Zip codes with lower median incomes (Table S1). A detailed analysis of the characteristics of patients hospitalized in Houston Methodist Hospital facilities in the two waves has recently been published (16).

10.1128/mBio.02707-20.7 TABLE S1 Patient demographics in wave 1 and wave 2. Download Table S1, PDF file, 0.03 MB. Copyright © 2020 Long et al. 2020 Long et al. This content is distributed under the terms of the Creative Commons Attribution 4.0 International license.

### SARS-CoV-2 genome sequencing and phylogenetic analysis.

To investigate the genomic architecture of the virus across the two waves, we sequenced the genomes of 5,085 SARS-CoV-2 strains dating to the earliest time of confirmed COVID-19 cases in Houston. Analysis of SARS-CoV-2 strains causing disease in the first wave (5 March through May 11) revealed the presence of many diverse virus genomes that, in the aggregate, represent the major clades identified globally to date (Fig. 1B). Clades G, GH, GR, and S were the four most abundantly represented phylogenetic groups (Fig. 1B). Strains with the Gly614 amino acid variant in spike protein represented 82% of the SARS-CoV-2 strains in wave 1 and 99.9% in wave 2 (*P < *0.0001; Fisher’s exact test) (Fig. 1B). This spike protein variant is characteristic of clades G, GH, and GR. Importantly, strains with the Gly614 variant represented only 71% of the specimens sequenced in March, the early part of wave 1 (Fig. 1B). We attribute the decrease in the number of strains with this variant observed in the first 2 weeks of March (Fig. 1B) to fluctuation caused by the relatively fewer COVID-19 cases occurring during that period.

### Relating spatiotemporal genome analysis with virus genotypes over two disease waves.

**FIG 2:**
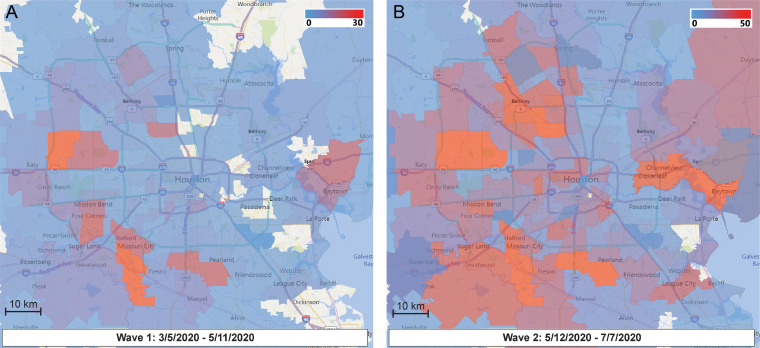
Sequential time-series heat maps for all COVID-19 Houston Methodist Hospital patients during the study period. The geospatial distribution of COVID-19 patients is based on Zip code. Panel A (left) shows the geospatial distribution of sequenced SARS-CoV-2 strains in wave 1, and panel B (right) shows the wave 2 distribution. The collection dates are shown at the bottom of each panel. The insets refer to numbers of strains in the color spectrum used. Note the differences in the numbers of strains presented in the panel A and panel B insets.

We examined the spatial and temporal mapping of genomic data to investigate community spread during wave 1 (Fig. 2). Rapid and widespread community dissemination occurred soon after the initial COVID-19 cases were reported in Houston. The heterogenous virus genotypes present very early in wave 1 indicate that multiple strains independently entered metropolitan Houston, rather than a single strain having been introduced and then spread. An important observation was that strains of most of the individual subclades were distributed over broad geographic areas (see Fig. S1 in the supplemental material). These findings are consistent with the known ability of SARS-CoV-2 to spread very rapidly from person to person.

10.1128/mBio.02707-20.1 FIG S1 Geographic distribution of representative SARS-CoV-2 subclades in the Houston metropolitan region. Blue-shaded areas denote Zip codes containing COVID-19 cases with the designated subclade. Download FIG S1, PDF file, 0.1 MB. Copyright © 2020 Long et al. 2020 Long et al. This content is distributed under the terms of the Creative Commons Attribution 4.0 International license.

### Relationship between virus clades, clinical characteristics of infected patients, and additional metadata.

It is possible that SARS-CoV-2 genome subtypes have different clinical characteristics, analogous to what is believed to have occurred with Ebola virus (17–19) and is known to occur for other pathogenic microbes (20). As an initial examination of this issue in SARS-CoV-2, we tested the hypothesis that patients with disease severe enough to warrant hospitalization were infected with a nonrandom subset of virus genotypes. We also examined the association between virus clades and disease severity based on overall mortality, highest level of required care (intensive care unit [ICU], intermediate care unit [IMU], inpatient or outpatient), need for mechanical ventilation, and length of stay. There was no simple relationship between virus clades and disease severity using these four indicators. Similarly, there was no simple relationship between virus clades and other metadata, such as sex, age, or ethnicity (Fig. S2).

10.1128/mBio.02707-20.2 FIG S2 Cladograms showing distribution of patient metadata, including (A) age (in decades), (B) sex, (C) ethnicity/ethnic group, (D) wave, (E) level of care, (F) mechanical ventilation, (G) length of stay, and (H) mortality. Download FIG S2, PDF file, 0.2 MB. Copyright © 2020 Long et al. 2020 Long et al. This content is distributed under the terms of the Creative Commons Attribution 4.0 International license.

### Machine learning analysis.

Machine learning models can be used to identify complex relationships not revealed by statistical analyses. We built machine learning models to test the hypothesis that virus genome sequence can predict patient outcomes, including mortality, length of stay, level of care, ICU admission, supplemental oxygen use, and mechanical ventilation. Models designed to predict outcomes based on virus genome sequence alone resulted in low F1 scores of less than 50% (0.41 to 0.49), and regression models showed similarly low *R*^2^ values (−0.01 to (−0.20) (Table S2). F1 scores near 50% are indicative of classifiers that are performing similarly to random chance. The use of patient metadata alone to predict patient outcome improved the model’s F1 scores by 5% to 10% (0.51 to 0.56) overall. The inclusion of patient metadata with virus genome sequence data improved most predictions of outcomes, compared to genome sequence alone, to 50% to 55% F1 overall (0.42 to 0.55) in the models (Table S2). The findings are indicative of two possibilities that are not mutually exclusive. First, patient metadata, such as age and sex, may provide more signal for the model to use and thus result in better accuracies. Second, the model’s use of single nucleotide polymorphisms (SNPs) may have resulted in overfitting. Most importantly, no SNP predicted a significant difference in outcome. A table of classifier accuracy scores and performance information is provided in Table S2.

10.1128/mBio.02707-20.8 TABLE S2 Classifier accuracy scores and performance of machine learning models. Download Table S2, PDF file, 0.03 MB. Copyright © 2020 Long et al. 2020 Long et al. This content is distributed under the terms of the Creative Commons Attribution 4.0 International license.

### Patient outcome and metadata correlations.

Overall, very few metadata categories correlated with patient outcomes (Table S3). Mortality was independently correlated with increasing age, with a Pearson correlation coefficient (PCC) equal to 0.27. This means that 27% of the variation in mortality can be predicted from patient age. Length of stay correlated independently with increasing age (PCC = 0.20). All other patient metadata correlations to outcomes had PCC values of less than 0.20 (Table S3).

10.1128/mBio.02707-20.9 TABLE S3 Pearson correlation coefficient data for correlation analysis. Download Table S3, PDF file, 0.04 MB. Copyright © 2020 Long et al. 2020 Long et al. This content is distributed under the terms of the Creative Commons Attribution 4.0 International license.

We further analyzed outcomes correlated to isolates from wave 1 and 2 and to the presence of the Gly614 variant in spike protein. Presence in wave 1 was independently correlated with mechanical ventilation days, overall length of stay, and ICU length of stay, with PCC values equal to 0.20, 0.18, and 0.14, respectively. Importantly, the presence of the Gly614 variant did not correlate with patient outcomes (Table S3).

### Analysis of the *nsp12* polymerase gene.

The SARS-CoV-2 genome encodes an RNA-dependent RNA polymerase (RdRp; also referred to as Nsp12) used in virus replication (21–24). Each of two amino acid substitutions (Phe479Leu and Val556Leu) in RdRp confers significant resistance *in vitro* to remdesivir, an adenosine analog (25). Remdesivir is inserted into RNA chains by RdRp during replication, resulting in premature termination of RNA synthesis and inhibition of virus replication. This compound has shown prophylactic and therapeutic benefits against Middle East respiratory syndrome coronavirus (MERS-CoV) and SARS-CoV-2 experimental infection in rhesus macaques (26, 27). Recent reports indicate that remdesivir has a therapeutic benefit in some patients with hospitalized COVID-19 (28–32), leading to its current widespread use in patients worldwide. Thus, it may be important to understand variation in RdRp in large collections of strain samples.

**FIG 3:**
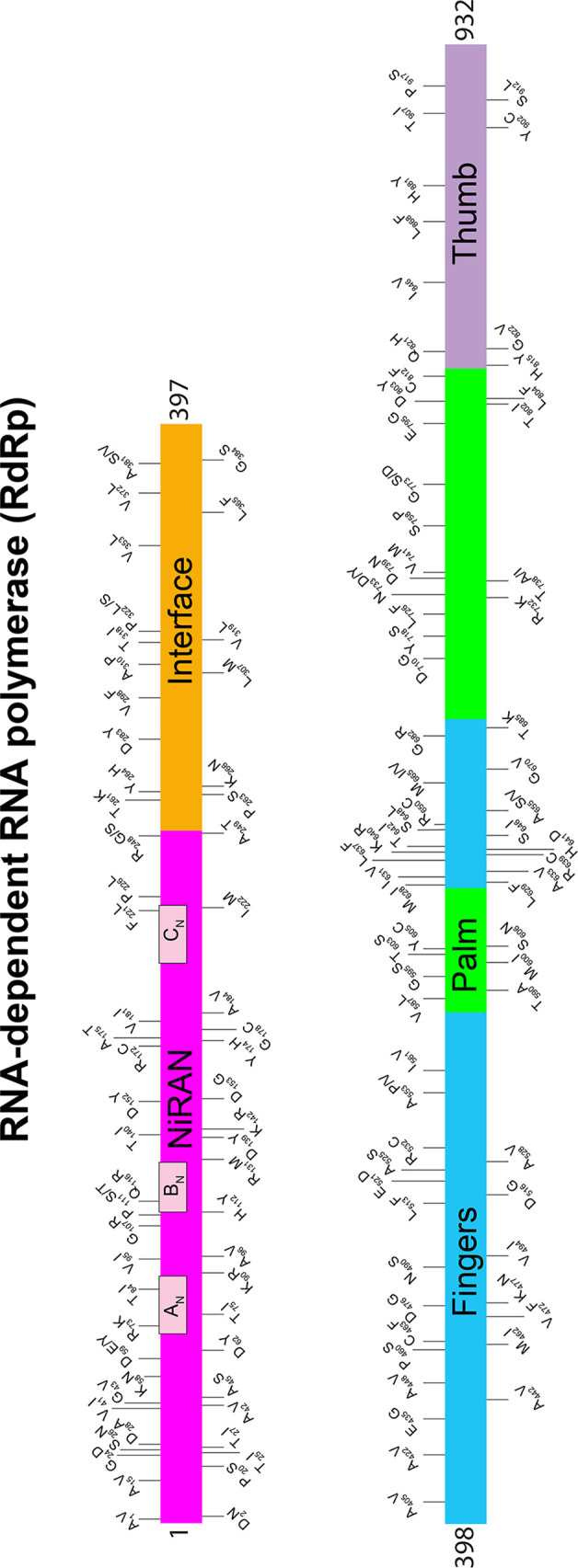
Location of amino acid replacements in RNA-dependent RNA polymerase (RdRp/Nsp12) among the 5,085 genomes of SARS-CoV-2 sequenced. The various RdRp domains are color coded. The numbers refer to amino acid sites. Note that several amino acid sites have multiple variants identified. The dates shown at the bottom of the figure panels represent month/day/year.

**FIG 4:**
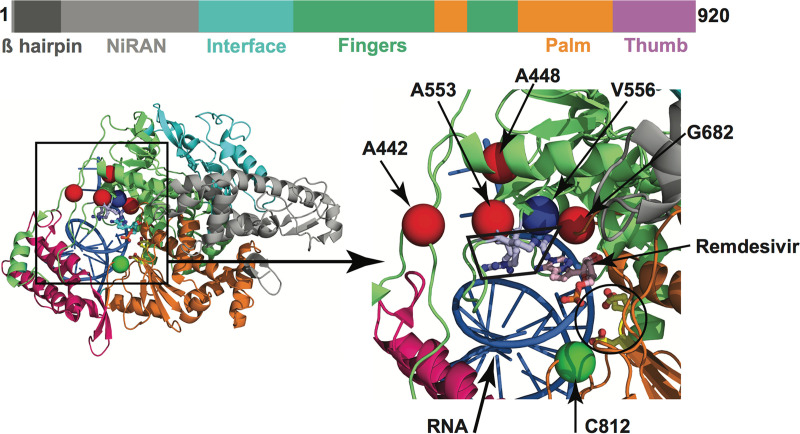
Amino acid changes identified in Nsp12 (RdRp) in this study that may influence interactions with remdesivir. The schematic at the top shows the domain architecture of Nsp12. (Left) Ribbon representation of the crystal structure of Nsp12-remdesivir monophosphate-RNA complex (PDB code: 7BV2). (Right) Magnified view of the boxed area in the left panel. The Nsp12 domains are colored as indicated in the schematic at the top. The catalytic site in Nsp12 is marked by a black circle at lower right in the right panel. The side chains of amino acids comprising the catalytic site of RdRp (Ser758, Asp759, and Asp760) are shown as balls and stick and colored yellow. The nucleotide binding site is boxed in the right panel. The side chains of amino acids participating in nucleotide binding (Lys544, Arg552, and Arg554) are shown as balls and sticks and colored light blue. A remdesivir molecule incorporated into the nascent RNA is shown as balls and sticks and colored light pink. The RNA is shown as a blue cartoon, and bases are shown as sticks. The positions of Cα atoms of amino acids identified in this study are shown as red and green spheres and labeled. The amino acids that are shown as red spheres are located above the nucleotide binding site, whereas Cys812 located at the catalytic site is shown as a green sphere. The side chain of active site residue Ser758 is shown as ball and sticks and colored yellow. The location of Cα atoms of remdesivir resistance-conferring amino acid Val556 is shown as a blue sphere and labeled.

To acquire data about allelic variation in the *nsp12* gene, we analyzed our 5,085 virus genomes. The analysis identified 265 SNPs, including 140 nonsynonymous (amino acid-altering) SNPs, resulting in amino acid replacements throughout the protein (Table 1) (Fig. 3 and 4; see also Fig. S3 and S4). The most common amino acid change was Pro322Leu, identified in 4,893 (96%) of the 5,085 patient isolates. This amino acid replacement is common in genomes from clades G, GH, and GR, which are distinguished from other SARS-CoV-2 clades by the presence of the Gly614 amino acid change in the spike protein. Most of the other amino acid changes in RdRp were present in relatively small numbers of strains, and some have been identified in other isolates in a publicly available database (33). Five prominent exceptions included the following amino acid replacements: Ala15Val in 138 strains, Met462Ile in 59 strains, Met600Ile in 75 strains, Thr907Ile in 45 strains, and Pro917Ser in 80 strains. All 75 Met600Ile strains were phylogenetically closely related members of clade G and also had the Pro322Leu amino acid replacement characteristic of this clade (Fig. S3). These data indicate that the Met600Ile change is likely the evolved state, derived from a precursor strain with the Pro322Leu replacement. Similarly, we investigated phylogenetic relationships among strains with the other four amino acid changes noted above. In all cases, the vast majority of strains with each amino acid replacement were found among individual subclades of strains (Fig. S3).

**TABLE 1:** Nonsynonymous SNPs of SARS-CoV-2 *nsp12*

Genomic locus	Gene locus	Amino acid change	Domain	No. of nonsynonymous SNPs		
				Wave 1 (*n* = 1,026)	Wave 2 (*n* = 4,059)	Total (*n* = 5,085)
13446	C3T	A1V	N terminus		2	2
13448	G5A	D2N	N terminus	1		1
13487	C44T	A15V	N terminus		138	138
13501	C58T	P20S	N terminus		1	1
13514	G71A	G24D	N terminus		3	3
13517	C74T	T25I	N terminus		4	4
13520	G77A	S26N	N terminus		1	1
13523	C80T	T27I	N terminus		1	1
13526	A83C	D28A	N terminus		1	1
13564	G121A	V41I	B hairpin		1	1
13568	C125T	A42V	B hairpin	1		1
13571	G128T	G43V	B hairpin	1		1
13576	G133T	A45S	B hairpin		12	12
13617	G174T	K58N	NiRAN^*a*^		1	1
13618	G175T	D59Y	NiRAN		24	24
13620	C177G	D59E	NiRAN		1	1
13627	G184T	D62Y	NiRAN	1		1
13661	G218A	R73K	NiRAN		1	1
13667	C224T	T75I	NiRAN		2	2
13694	C251T	T84I	NiRAN		1	1
13712	A269G	K90R	NiRAN		1	1
13726	G283A	V95I	NiRAN		1	1
13730	C287T	A96V	NiRAN	2	2	4
13762	G319C	G107R	NiRAN		1	1
13774	C331A	P111T	NiRAN		1	1
13774	C331T	P111S	NiRAN		15	15
13777	C334T	H112Y	NiRAN		1	1
13790	A347G	Q116R	NiRAN		2	2
13835	G392T	R131M	NiRAN		1	1
13858	G415T	D139Y	NiRAN		3	3
13862	C419T	T140I	NiRAN	1	5	6
13868	A425G	K142R	NiRAN	1		1
13897	G454T	D152Y	NiRAN		4	4
13901	A458G	D153G	NiRAN		2	2
13957	C514T	R172C	NiRAN		2	2
13963	T520C	Y174H	NiRAN		1	1
13966	G523A	A175T	NiRAN		1	1
13975	G532T	G178C	NiRAN		4	4
13984	G541A	V181I	NiRAN		1	1
13994	C551T	A184V	NiRAN		8	8
14104	T661C	F221L	NiRAN	2		2
14109	A666G	I222M	NiRAN	1		1
14120	C677T	P226L	NiRAN		2	2
14185	A742G	R248G	NiRAN		1	1
14187	G744T	R248S	NiRAN		1	1
14188	G745A	A249T	NiRAN		1	1
14225	C782A	T261K	Interface		4	4
14230	C787T	P263S	Interface		1	1
14233	T790C	Y264H	Interface		1	1
14241	G798T	K266N	Interface		1	1
14290	G847T	D283Y	Interface		1	1
14335	G892T	V298F	Interface		8	8
14362	C919A	L307M	Interface		2	2
14371	G928C	A310P	Interface	1		1
14396	C953T	T318I	Interface		1	1
14398	G955T	V319L	Interface		1	1
14407	C964T	P322S	Interface		2	2
14408	C965T	P322L	Interface	843	4,050	4,893
14500	G1057T	V353L	Interface		5	5
14536	C1093T	L365F	Interface		1	1
14557	G1114T	V372L	Fingers		4	4
14584	G1141T	A381S	Fingers		1	1
14585	C1142T	A381V	Fingers		10	10
14593	G1150A	G384S	Fingers	1		1
14657	C1214T	A405V	Fingers		1	1
14708	C1265T	A422V	Fingers		1	1
14747	A1304G	E435G	Fingers		2	2
14768	C1325T	A442V	Fingers		21	21
14786	C1343T	A448V	Fingers	3	6	9
14821	C1378T	P460S	Fingers		1	1
14829	G1386T	M462I	Fingers		59	59
14831	G1388T	C463F	Fingers		3	3
14857	G1414T	V472F	Fingers		1	1
14870	A1427G	D476G	Fingers		5	5
14874	G1431T	K477N	Fingers	1		1
14912	A1469G	N490S	Fingers	1	1	2
14923	G1480A	V494I	Fingers		2	2
14980	C1537T	L513F	Fingers	1	1	2
14990	A1547G	D516G	Fingers		1	1
15006	G1563C	E521D	Fingers	2	3	5
15016	G1573T	A525S	Fingers		3	3
15026	C1583T	A528V	Fingers	5	1	6
15037	C1594T	R532C	Fingers		1	1
15100	G1657C	A553P	Fingers		1	1
15101	C1658T	A553V	Fingers		1	1
15124	A1681G	I561V	Fingers		2	2
15202	G1759C	V587L	Palm		7	7
15211	A1768G	T590A	Palm		1	1
15226	G1783A	G595S	Palm		1	1
15243	G1800T	M600I	Palm	71	4	75
15251	C1808G	T603S	Palm		1	1
15257	A1814G	Y605C	Palm		1	1
15260	G1817A	S606N	Palm		1	1
15327	G1884T	M628I	Fingers	3	1	4
15328	C1885T	L629F	Fingers	1		1
15334	A1891G	I631V	Fingers		1	1
15341	C1898T	A633V	Fingers		1	1
15352	C1909T	L637F	Fingers		1	1
15358	C1915T	R639C	Fingers		1	1
15362	A1919G	K640R	Fingers		1	1
15364	C1921G	H641D	Fingers	1		1
15368	C1925T	T642I	Fingers	1		1
15380	G1937T	S646I	Fingers	1		1
15386	C1943T	S648L	Fingers		2	2
15391	C1948T	R650C	Fingers		1	1
15406	G1963T	A655S	Fingers		3	3
15407	C1964T	A655V	Fingers	1		1
15436	A1993G	M665V	Fingers		2	2
15438	G1995T	M665I	Fingers		24	24
15452	G2009T	G670V	Fingers		28	28
15487	G2044C	G682R	Palm		1	1
15497	C2054A	T685K	Palm		1	1
15572	A2129G	D710G	Palm		1	1
15596	A2153G	Y718S	Palm	2		2
15619	C2176T	L726F	Palm	1		1
15638	G2195A	R732K	Palm	1		1
15640	A2197G	N733D	Palm	1		1
15640	A2197T	N733Y	Palm	1		1
15655	A2212G	T738A	Palm		2	2
15656	C2213T	T738I	Palm		2	2
15658	G2215A	D739N	Palm		2	2
15664	G2221A	V741M	Palm		1	1
15715	T2272C	S758P	Palm		1	1
15760	G2317A	G773S	Palm	1		1
15761	G2318A	G773D	Palm		1	1
15827	A2384G	E795G	Palm	1		1
15848	C2405T	T802I	Palm		1	1
15850	G2407T	D803Y	Palm		1	1
15853	C2410T	L804F	Palm		2	2
15878	G2435T	C812F	Palm		1	1
15886	C2443T	H815Y	Palm		1	1
15906	G2463T	Q821H	Thumb	1	1	2
15908	G2465T	G822V	Thumb		1	1
15979	A2536G	I846V	Thumb	4		4
16045	C2602T	L868F	Thumb		1	1
16084	C2641T	H881Y	Thumb		1	1
16148	A2705G	Y902C	Thumb		1	1
16163	C2720T	T907I	Thumb		45	45
16178	C2735T	S912L	Thumb		2	2
16192	C2749T	P917S	Thumb		80	80

a NiRAN, nucleotidyl transferase domain.

10.1128/mBio.02707-20.3 FIG S3 Distribution of subclades characterized by particular amino acid replacements in Nsp12 (RdRp). Download FIG S3, PDF file, 0.1 MB. Copyright © 2020 Long et al. 2020 Long et al. This content is distributed under the terms of the Creative Commons Attribution 4.0 International license.

10.1128/mBio.02707-20.4 FIG S4 Mapping the location of amino acid replacements on Nsp12 (RdRp) from COVID-19 virus. The schematic on the top shows the domain architecture of Nsp12. The individual domains of Nsp12 are color coded and labeled. A ribbon representation of the crystal structure of an Nsp12-remdesivir monophosphate-RNA complex is shown (PDB code: 7BV2). The structure in the right panel was obtained by rotating the left panel 180° along the *y* axis. The Nsp12 domains are colored as shown in the schematic at the top. The positions of Cα atoms of the surface-exposed amino acids identified in this study are shown as yellow spheres, whereas the positions of Cα atoms of the buried amino acids are depicted as cyan spheres. The catalytic site in RdRp is marked by a black circle in the right panel. The side chains of amino acids comprising the catalytic site of RdRp are shown as balls and sticks and colored yellow. The nucleotide binding site is boxed and labeled in the right panel. The side chains of amino acids participating in nucleotide binding (Lys545, Arg553, and Arg555) are shown as balls and sticks. Remdesivir molecules incorporated into the nascent RNA are shown as balls and sticks and colored light pink. The RNA is shown as a blue cartoon, and bases are shown as sticks. The positions of Cα atoms of amino acids that are predicted to influence remdesivir binding are shown as red spheres. Amino acid Cys812 located at the catalytic site is shown as green sphere. The location of Cα atoms corresponding to remdesivir resistance-conferring amino acid Val556 is shown as a blue sphere and labeled. Download FIG S4, PDF file, 0.6 MB. Copyright © 2020 Long et al. 2020 Long et al. This content is distributed under the terms of the Creative Commons Attribution 4.0 International license.

Importantly, none of the observed amino acid polymorphisms in RdRp were located precisely at two sites known to cause *in vitro* resistance to remdesivir (25). Most of the amino acid changes were found to be located distantly from the RNA binding and catalytic sites (Fig. S4; see also Table 1). However, replacements at six amino acid residues (Ala442Val, Ala448Val, Ala553Pro/Val, Gly682Arg, Ser758Pro, and Cys812Phe) may potentially interfere with either remdesivir binding or RNA synthesis. Four (Ala442Val, Ala448Val, Ala553Pro/Val, and Gly682Arg) of the six substitution sites are located immediately above the nucleotide-binding site, which is comprised of Lys544, Arg552, and Arg554 residues as shown by structural studies (Fig. 4). The positions of these four variant amino acid sites are comparable to that of Val556 (Fig. 4), and a Val556Leu mutation in SARS-CoV was identified to confer resistance to remdesivir *in vitro* (25). The other two substitutions (Ser758Pro and Cys812Phe) are inferred to be located either at, or in the immediate proximity of, the catalytic active site, which is comprised of three contiguous residues (Ser758, Asp759, and Asp760). A proline substitution that we identified at Ser758 (Ser758Pro) is likely to negatively impact RNA synthesis. Although Cys812 is not directly involved in the catalysis of RNA synthesis, it is only 3.5 Å away from Asp760. The introduction of the bulkier phenylalanine substitution at Cys812 (Cys812Phe) may impair RNA synthesis. Consequently, these two substitutions are expected to detrimentally affect virus replication or fitness.

### Analysis of the gene encoding the spike protein.

**FIG 5:**
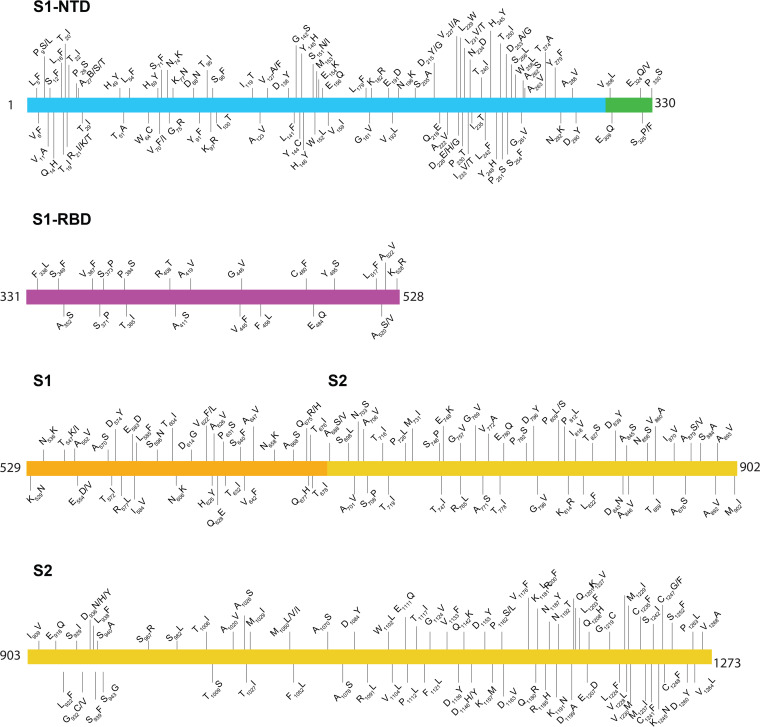
Locations of amino acid replacements in spike protein among the 5,085 genomes of SARS-CoV-2 sequenced. The various spike protein domains are color coded. The numbers refer to amino acid sites. Note that many amino acid sites have multiple variants identified.

The densely glycosylated spike protein of SARS-CoV-2 and of its close coronavirus relatives binds directly to host cell angiotensin-converting enzyme 2 (ACE2) receptors to enter host cells (34–36). Thus, the spike protein is a major translational research target, including intensive vaccine and therapeutic antibody research (34–63). Analysis of the gene encoding the spike protein identified 470 SNPs, including 285 that produce amino acid changes (Table 2; see also Fig. 5). Forty-nine of these replacements (V11A, T51A, W64C, I119T, E156Q, S205A, D228G, L229W, P230T, N234D, I235T, T274A, A288V, E324Q, E324V, S325P, S349F, S371P, S373P, T385I, A419V, C480F, Y495S, L517F, K528R, Q628E, T632I, S708P, T719I, P728L, S746P, E748K, G757V, V772A, K814R, D843N, S884A, M902I, I909V, E918Q, S982L, M1029I, Q1142K, K1157M, Q1180R, D1199A, C1241F, C1247G, and V1268A) were not represented in a publicly available database (33) as of 19 August 2020. Interestingly, 25 amino acid sites have three distinct variants (that is, the reference amino acid plus two additional variant amino acids), and 5 amino acid sites (amino acid positions 21, 27, 228, 936, and 1050) have four distinct variants represented in our sample of 5,085 genomes (Table 2; see also Fig. 5).

**TABLE 2:** Nonsynonymous SNPs in SARS-CoV-2 spike protein^*a*^

Genomic locus	Gene locus	Amino acid change	Domain	No. of nonsynonymous SNPs		
				Wave 1 (*n* = 1,026)	Wave 2 (*n* = 4,059)	Total (*n* = 5,085)
21575	C13T	L5F	S1	11	25	36
21578	G16T	V6F	S1		1	1
21587	C25T	P9S	S1	2		2
21588	C26T	P9L	S1	1	1	2
21594	T32C	V11A	S1		1	1
21597	C35T	S12F	S1		6	6
21604	G42T	Q14H	S1		1	1
21614	C52T	L18F	S2—NTD	1	11	12
21618	C56T	T19I	S2—NTD	1	1	2
21621	C59T	T20I	S2—NTD		1	1
21624	G62T	R21I	S2—NTD		6	6
21624	G62A	R21K	S2—NTD		1	1
21624	G62C	R21T	S2—NTD		3	3
21627	C65T	T22I	S2—NTD	2	4	6
21638	C76T	P26S	S2—NTD		17	17
21641	G79T	A27S	S2—NTD	1	1	2
21641	G79A	A27T	S2—NTD	1		1
21642	C80T	A27V	S2—NTD		1	1
21648	C86T	T29I	S2—NTD	1	4	5
21707	C145T	H49Y	S2—NTD		142	142
21713	A151G	T51A	S2—NTD		1	1
21724	G162T	L54F	S2—NTD		11	11
21754	G192T	W64C	S2—NTD		1	1
21767	C205T	H69Y	S2—NTD	1	7	8
21770	G208A	V70I	S2—NTD		1	1
21770	G208T	V70F	S2—NTD	1		1
21774	C212T	S71F	S2—NTD		1	1
21784	T222A	N74K	S2—NTD	1		1
21785	G223C	G75R	S2—NTD		1	1
21793	G231T	K77N	S2—NTD		1	1
21824	G262A	D88N	S2—NTD		1	1
21834	A272T	Y91F	S2—NTD		1	1
21846	C284T	T95I	S2—NTD	1	10	11
21852	A290G	K97R	S2—NTD		1	1
21855	C293T	S98F	S2—NTD	1	2	3
21861	T299C	I100T	S2—NTD		2	2
21918	T356C	I119T	S2—NTD	1		1
21930	C368T	A123V	S2—NTD		1	1
21941	G379T	V127F	S2—NTD		1	1
21942	T380C	V127A	S2—NTD		4	4
21974	G412T	D138Y	S2—NTD	2		2
21985	G423T	L141F	S2—NTD		1	1
21986	G424A	G142S	S2—NTD		2	2
21993	A431G	Y144C	S2—NTD	1		1
21995	T433C	Y145H	S2—NTD	2		2
21998	C436T	H146Y	S2—NTD	1	2	3
22014	G452A	S151N	S2—NTD		1	1
22014	G452T	S151I	S2—NTD		2	2
22017	G455T	W152L	S2—NTD	1	1	2
22021	G459T	M153I	S2—NTD		1	1
22021	G459A	M153I	S2—NTD		1	1
22022	G460A	E154K	S2—NTD		1	1
22028	G466C	E156Q	S2—NTD	2		2
22037	G475A	V159I	S2—NTD	1		1
22097	C535T	L179F	S2—NTD		1	1
22104	G542T	G181V	S2—NTD		1	1
22107	A545G	K182R	S2—NTD		1	1
22135	A573T	E191D	S2—NTD		1	1
22139	G577T	V193L	S2—NTD		1	1
22150	T588G	N196K	S2—NTD	1		1
22175	T613G	S205A	S2—NTD		1	1
22205	G643T	D215Y	S2—NTD		1	1
22206	A644G	D215G	S2—NTD		2	2
22214	C652G	Q218E	S2—NTD		1	1
22227	C665T	A222V	S2—NTD		1	1
22241	G679A	V227I	S2—NTD		2	2
22242	T680C	V227A	S2—NTD	1		1
22244	G682C	D228H	S2—NTD		2	2
22245	A683G	D228G	S2—NTD	1		1
22246	T684G	D228E	S2—NTD	2		2
22248	T686G	L229W	S2—NTD	1		1
22250	C688A	P230T	S2—NTD	1		1
22253	A691G	I231V	S2—NTD	1		1
22254	T692C	I231T	S2—NTD	1		1
22259	A697G	I233V	S2—NTD	1		1
22260	T698C	I233T	S2—NTD	1		1
22262	A700G	N234D	S2—NTD	1		1
22266	T704C	I235T	S2—NTD	1		1
22281	C719T	T240I	S2—NTD		5	5
22286	C724T	L242F	S2—NTD		1	1
22295	C733T	H245Y	S2—NTD		2	2
22304	T742C	Y248H	S2—NTD		3	3
22311	C749T	T250I	S2—NTD	1	4	5
22313	C751T	P251S	S2—NTD		2	2
22320	A758G	D253G	S2—NTD		2	2
22320	A758C	D253A	S2—NTD	1		1
22323	C761T	S254F	S2—NTD		3	3
22329	C767T	S256L	S2—NTD	1		1
22335	G773T	W258L	S2—NTD	1		1
22344	G782T	G261V	S2—NTD	3		3
22346	G784T	A262S	S2—NTD		4	4
22350	C788T	A263V	S2—NTD	1		1
22382	A820G	T274A	S2—NTD		1	1
22398	A836T	Y279F	S2—NTD	1		1
22408	T846G	N282K	S2—NTD		1	1
22425	C863T	A288V	S2—NTD		1	1
22430	G868T	D290Y	S2—NTD	1		1
22484	G922T	V308L	S1		3	3
22487	G925C	E309Q	S1		1	1
22532	G970C	E324Q	S1		1	1
22533	A971T	E324V	S1		1	1
22535	T973C	S325P	S1		1	1
22536	C974T	S325F	S1		1	1
22550	C988T	P330S	S2—RBD		2	2
22574	T1012C	F338L	S2—RBD	1		1
22608	C1046T	S349F	S2—RBD		1	1
22616	G1054T	A352S	S2—RBD		7	7
22661	G1099T	V367F	S2—RBD		1	1
22673	T1111C	S371P	S2—RBD		3	3
22679	T1117C	S373P	S2—RBD		1	1
22712	C1150T	P384S	S2—RBD		1	1
22716	C1154T	T385I	S2—RBD	3		3
22785	G1223C	R408T	S2—RBD		1	1
22793	G1231T	A411S	S2—RBD		1	1
22818	C1256T	A419V	S2—RBD	1		1
22895	G1333T	V445F	S2—RBD		1	1
22899	G1337T	G446V	S2—RBD	2		2
22928	T1366C	F456L	S2—RBD	1		1
23001	G1439T	C480F	S2—RBD		1	1
23012	G1450C	E484Q	S2—RBD	1		1
23046	A1484C	Y495S	S2—RBD		1	1
23111	C1549T	L517F	S2—RBD		1	1
23120	G1558T	A520S	S2—RBD	1	6	7
23121	C1559T	A520V	S2—RBD		1	1
23127	C1565T	A522V	S2—RBD	1	1	2
23145	A1583G	K528R	S2—RBD		2	2
23149	G1587T	K529N	S1		1	1
23170	C1608A	N536K	S1		1	1
23202	C1640A	T547K	S1		2	2
23202	C1640T	T547I	S1		1	1
23223	A1661T	E554V	S1		2	2
23224	G1662T	E554D	S1	4	31	35
23270	G1708T	A570S	S1		3	3
23277	C1715T	T572I	S1	5	5	10
23282	G1720T	D574Y	S1		1	1
23292	G1730T	R577L	S1	1		1
23311	G1749T	E583D	S1		6	6
23312	A1750G	I584V	S1		1	1
23315	C1753T	L585F	S1	1	7	8
23349	G1787A	S596N	S1		1	1
23373	C1811T	T604I	S1		2	2
23380	C1818A	N606K	S1		2	2
23403	A1841G	D614G	S1	841	4,054	4,895
23426	G1864T	V622F	S1		2	2
23426	G1864C	V622L	S1		2	2
23435	C1873T	H625Y	S1		1	1
23439	C1877T	A626V	S1		1	1
23444	C1882G	Q628E	S1		7	7
23453	C1891T	P631S	S1	1		1
23457	C1895T	T632I	S1		1	1
23481	C1919T	S640F	S1	1	42	43
23486	G1924T	V642F	S1		1	1
23502	C1940T	A647V	S1		1	1
23536	C1974A	N658K	S1		4	4
23564	G2002T	A668S	S1		1	1
23586	A2024G	Q675R	S1		14	14
23587	G2025C	Q675H	S1		1	1
23587	G2025T	Q675H	S1		4	4
23589	C2027T	T676I	S1	1	2	3
23593	G2031T	Q677H	S1	1	1	2
23595	C2033T	T678I	S1	1		1
23624	G2062T	A688S	S2		4	4
23625	C2063T	A688V	S2		16	16
23655	C2093T	S698L	S2		1	1
23664	C2102T	A701V	S2		21	21
23670	A2108G	N703S	S2		1	1
23679	C2117T	A706V	S2		1	1
23684	T2122C	S708P	S2		1	1
23709	C2147T	T716I	S2		1	1
23718	C2156T	T719I	S2		1	1
23745	C2183T	P728L	S2	1		1
23755	G2193T	M731I	S2	3	1	4
23798	T2236C	S746P	S2		1	1
23802	C2240T	T747I	S2		1	1
23804	G2242A	E748K	S2		1	1
23832	G2270T	G757V	S2		1	1
23856	G2294T	R765L	S2		1	1
23868	G2306T	G769V	S2		3	3
23873	G2311T	A771S	S2		8	8
23877	T2315C	V772A	S2		1	1
23895	C2333T	T778I	S2		1	1
23900	G2338C	E780Q	S2		1	1
23936	C2374T	P792S	S2		1	1
23948	G2386T	D796Y	S2		2	2
23955	G2393T	G798V	S2	1		1
23987	C2425T	P809S	S2		2	2
23988	C2426T	P809L	S2		1	1
23997	C2435T	P812L	S2		1	1
24003	A2441G	K814R	S2		1	1
24014	A2452G	I818V	S2—FP		5	5
24026	C2464T	L822F	S2—FP		97	97
24041	A2479T	T827S	S2—FP		4	4
24077	G2515T	D839Y	S2	2		2
24089	G2527A	D843N	S2	1	1	2
24095	G2533T	A845S	S2		5	5
24099	C2537T	A846V	S2		1	1
24129	A2567G	N856S	S2		7	7
24138	C2576T	T859I	S2		5	5
24141	T2579C	V860A	S2		1	1
24170	A2608G	I870V	S2		3	3
24188	G2626T	A876S	S2		1	1
24197	G2635T	A879S	S2		31	31
24198	C2636T	A879V	S2		1	1
24212	T2650G	S884A	S2		11	11
24237	C2675T	A892V	S2		1	1
24240	C2678T	A893V	S2	1		1
24268	G2706T	M902I	S2		1	1
24287	A2725G	I909V	S2—HR1		2	2
24314	G2752C	E918Q	S2—HR1	1		1
24328	G2766C	L922F	S2—HR1		2	2
24348	G2786T	S929I	S2—HR1		1	1
24356	G2794T	G932C	S2—HR1		1	1
24357	G2795T	G932V	S2—HR1		1	1
24368	G2806A	D936N	S2—HR1		3	3
24368	G2806C	D936H	S2—HR1		1	1
24368	G2806T	D936Y	S2—HR1	3	4	7
24374	C2812T	L938F	S2—HR1		3	3
24378	C2816T	S939F	S2—HR1		4	4
24380	T2818G	S940A	S2—HR1		5	5
24389	A2827G	S943G	S2—HR1		6	6
24463	C2901A	S967R	S2—HR1	2		2
24507	C2945T	S982L	S2—HR1		1	1
24579	C3017T	T1006I	S2—CH		1	1
24588	C3026G	T1009S	S2—CH		1	1
24621	C3059T	A1020V	S2—CH	1		1
24638	G3076T	A1026S	S2—CH		2	2
24642	C3080T	T1027I	S2—CH	5		5
24649	G3087T	M1029I	S2—CH		1	1
24710	A3148T	M1050L	S2		1	1
24710	A3148G	M1050V	S2	1	1	2
24712	G3150T	M1050I	S2		2	2
24718	C3156A	F1052L	S2	1	166	167
24770	G3208T	A1070S	S2		2	2
24794	G3232T	A1078S	S2—CD	3	2	5
24812	G3250T	D1084Y	S2—CD	1	29	30
24834	G3272T	R1091L	S2—CD	1		1
24867	G3305T	W1102L	S2—CD		1	1
24872	G3310T	V1104L	S2—CD		1	1
24893	G3331C	E1111Q	S2—CD		2	2
24897	C3335T	P1112L	S2—CD	2	2	4
24912	C3350T	T1117I	S2—CD		1	1
24923	T3361C	F1121L	S2—CD		2	2
24933	G3371T	G1124V	S2—CD	1	2	3
24959	G3397T	V1133F	S2—CD		1	1
24977	G3415T	D1139Y	S2—CD		1	1
24986	C3424A	Q1142K	S2	1		1
24998	G3436T	D1146Y	S2		4	4
24998	G3436C	D1146H	S2		13	13
25019	G3457T	D1153Y	S2		11	11
25032	A3470T	K1157M	S2	1		1
25046	C3484T	P1162S	S2		5	5
25047	C3485T	P1162L	S2		3	3
25050	A3488T	D1163V	S2		2	2
25088	G3526T	V1176F	S2		18	18
25101	A3539G	Q1180R	S2		1	1
25104	A3542G	K1181R	S2		4	4
25116	G3554A	R1185H	S2		2	2
25121	A3559T	N1187Y	S2		1	1
25135	G3573T	K1191N	S2		1	1
25137	A3575C	N1192T	S2	1		1
25158	A3596C	D1199A	S2		1	1
25160	C3598T	L1200F	S2		1	1
25163	C3601A	Q1201K	S2		1	1
25169	C3607T	L1203F	S2	1		1
25183	G3621T	E1207D	S2		1	1
25186	G3624T	Q1208H	S2	1		1
25217	G3655T	G1219C	S2	1	3	4
25234	G3672T	L1224F	S2		1	1
25241	A3679G	I1227V	S2	1		1
25244	G3682T	V1228L	S2		2	2
25249	G3687T	M1229I	S2		1	1
25249	G3687C	M1229I	S2		2	2
25250	G3688A	V1230M	S2		1	1
25266	G3704T	C1235F	S2		4	4
25273	G3711T	M1237I	S2		2	2
25284	G3722T	C1241F	S2		1	1
25287	G3725T	S1242I	S2		4	4
25297	G3735T	K1245N	S2		1	1
25301	T3739G	C1247G	S2		1	1
25302	G3740T	C1247F	S2		4	4
25305	G3743T	C1248F	S2		2	2
25317	C3755T	S1252F	S2		1	1
25340	G3778T	D1260Y	S2		2	2
25350	C3788T	P1263L	S2	1	2	3
25352	G3790T	V1264L	S2		1	1
25365	T3803C	V1268A	S2		1	1

a The domain region of RBD is based on structural information published previously by Cai et al. (93). Forty-nine of these amino acid replacements (V11A, T51A, W64C, I119T, E156Q, S205A, D228G, L229W, P230T, N234D, I235T, T274A, A288V, E324Q, E324V, S325P, S349F, S371P, S373P, T385I, A419V, C480F, Y495S, L517F, K528R, Q628E, T632I, S708P, T719I, P728L, S746P, E748K, G757V, V772A, K814R, D843N, S884A, M902I, I909V, E918Q, S982L, M1029I, Q1142K, K1157M, Q1180R, D1199A, C1241F, C1247G, and V1268A) were not represented in a publicly available database (33) as of 19 August 2020.

**FIG 6:**
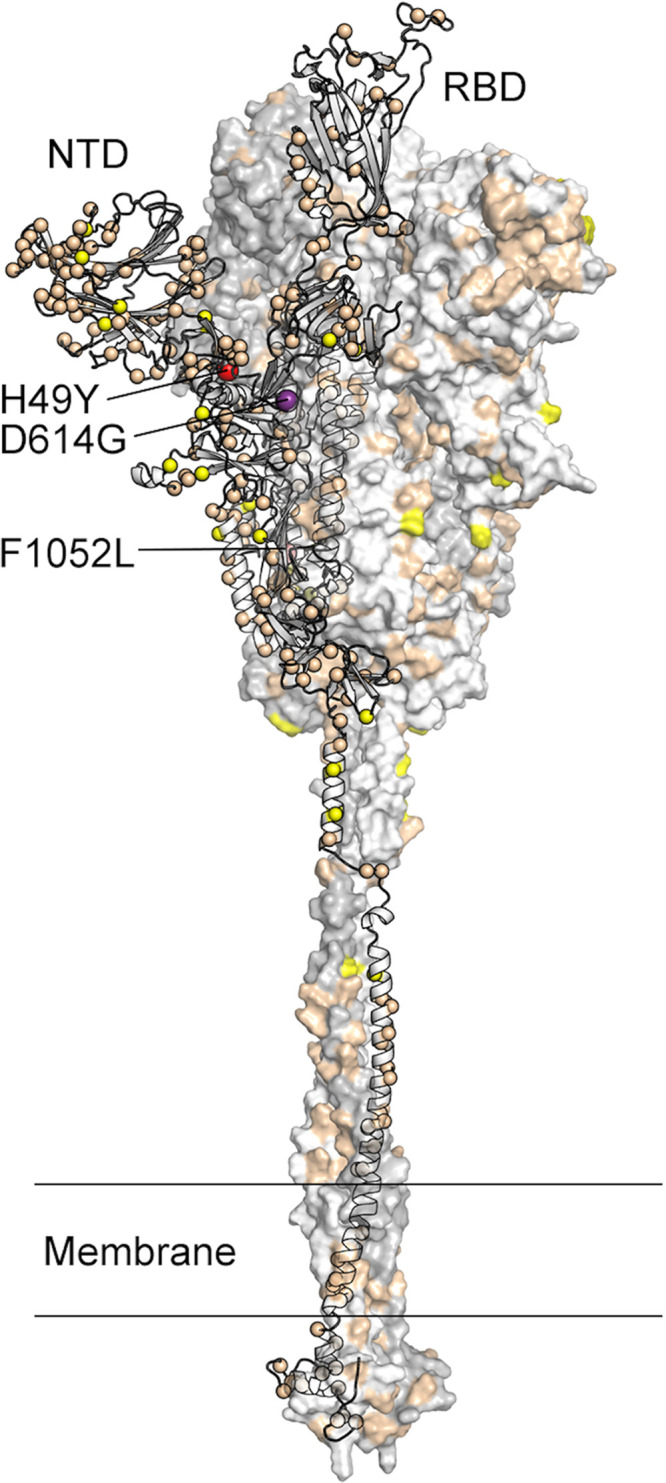
Location of amino acid substitutions mapped on the SARS-CoV-2 spike protein. The figure presents a model of the SARS-CoV-2 spike protein with one protomer shown as ribbons and the other two protomers shown as a molecular surface. The Cα atom of residues found to be substituted in one or more virus isolates identified in this study is shown as a sphere on the ribbon representation. Residues found to be substituted in 1 to 9 isolates are colored tan, those substituted in 10 to 99 isolates yellow, those substituted in 100 to 999 isolates red (H49Y and F1052L), and those substituted in >1,000 isolates purple (D614G). The surface of the amino-terminal domain (NTD) that is distal to the trimeric axis has a high density of substituted residues. RBD, receptor binding domain.

We mapped the location of amino acid replacements onto a model of the full-length spike protein (34, 64) and observed that the substitutions are found in each subunit and domain of the spike (Fig. 6). However, the distribution of amino acid changes is not uniform throughout the protein regions. For example, compared to some other regions of the spike protein, the RBD has relatively few amino acid changes, and the frequency of strains with these substitutions is low, each occurring in fewer than 10 isolates. This finding is consistent with the functional constraints on RBD to mediate interaction with ACE2. In contrast, the periphery of the S1 subunit amino-terminal domain (NTD) contains a dense cluster of substituted residues, with some single amino acid replacements found in 10 to 20 isolates (Table 2; see also Fig. 5 and 6). Clustering of amino acid changes in a distinct region of the spike protein may be a signal of positive selection. Inasmuch as infected patients make antibodies against the NTD, we favor the idea that host immune selection is among the forces contributing to some of the amino acid variation in this region. One NTD substitution, H49Y, was found in 142 isolates. This position is not well exposed on the surface of the NTD and likely does not represent a result of immune pressure. The same is true for another highly represented substitution, F1052L. This substitution was observed in 167 isolates, and F1052 is buried within the core of the S2 subunit. The substitution observed most frequently in the spike protein in our sample is D614G, a change observed in 4,895 of the isolates. As noted above, strains with the Gly614 variant significantly increased in frequency in wave 2 compared to wave 1.

As observed with RdRp, the majority of strains with each single amino acid change in the spike protein were found on a distinct phylogenetic lineage (Fig. S5), indicating identity by descent. A prominent exception is the Leu5Phe replacement that is present in all major clades, suggesting that this amino acid change arose multiple times independently or very early in the course of SARS-CoV-2 evolution. Finally, we note that examination of the phylogenetic distribution of strains with multiple distinct amino acid replacements at the same site (e.g., Arg21Ile/Lys/Thr, Ala27Ser/Thr/Val, etc.) revealed that they were commonly found in different genetic branches, consistent with independent origin (Fig. S5).

10.1128/mBio.02707-20.5 FIG S5 Distribution of subclades characterized by particular amino acid replacements in spike protein. Download FIG S5, PDF file, 0.5 MB. Copyright © 2020 Long et al. 2020 Long et al. This content is distributed under the terms of the Creative Commons Attribution 4.0 International license.

### Cycle threshold (*C_T_*) comparison of SARS-CoV-2 strains with either the Asp614 or Gly614 amino acid replacements in spike protein.

**FIG 7:**
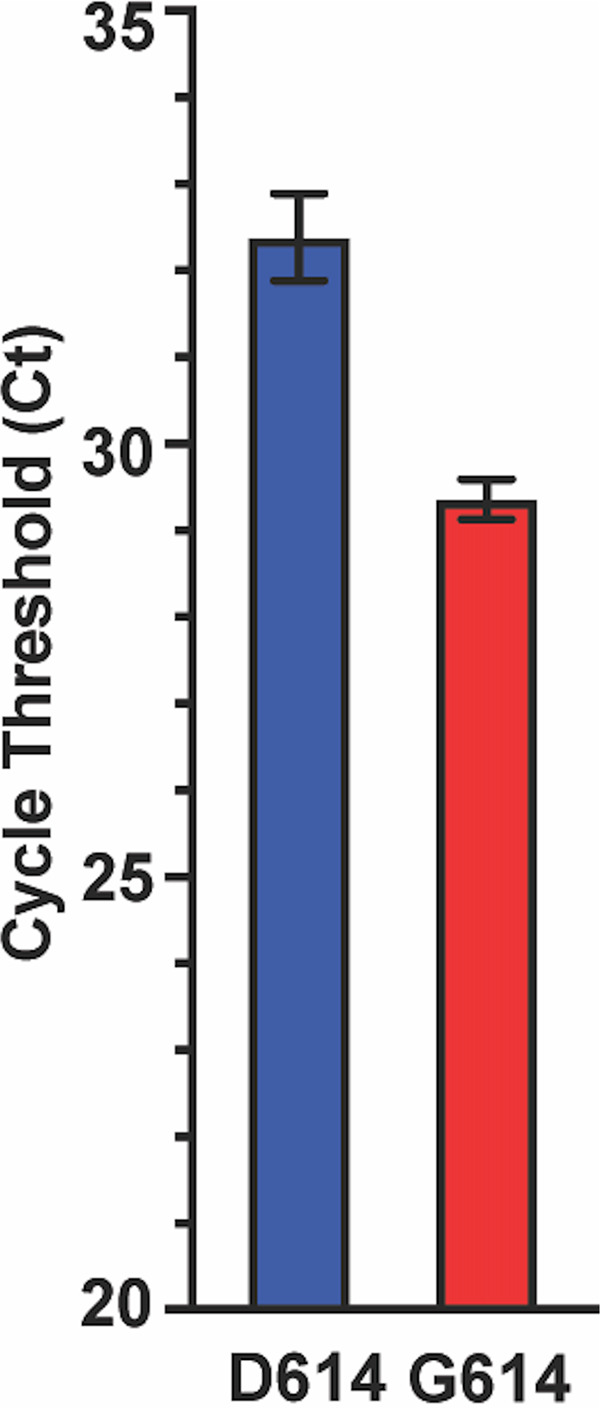
Cycle threshold (*C_T_*) data for every SARS-CoV-2 patient sample tested using the Hologic Panther assay. Data are presented as means ± standard errors of the means for strains with an aspartate (D614, *n *= 102 strains, blue) or glycine (G614, *n *= 812 strains, red) at amino acid 614 of the spike protein. Mann-Whitney test; *, *P < *0.0001.

It has been reported that patients infected with strains having the spike protein Gly614 variant have, on average, higher virus loads on initial diagnosis (65–69). To determine if this is the case in Houston strains, we examined the cycle threshold (*C_T_*) for every sequenced strain that was detected from a patient specimen using the SARS-CoV-2 assay done by the use of a Hologic Panther instrument. We identified a significant difference (*P < *0.0001) between the mean *C_T_* values determined for strains with an Asp614 (*n *= 102) or Gly614 (*n *= 812) variant of the spike protein (Fig. 7). Strains with Gly614 had a *C_T_* value significantly lower than that calculated for strains with the Asp614 variant, indicating that the patients infected with the Gly614 strains had, on average, higher virus loads on initial diagnosis than the patients infected by strains with the Asp614 variant (Fig. 7). This observation is consistent with the conjecture that, on average, strains with the Gly614 variant are better able to disseminate (65–69).

### Characterization of recombinant proteins with single amino acid replacements in the receptor binding domain region of spike protein.

**FIG 8:**
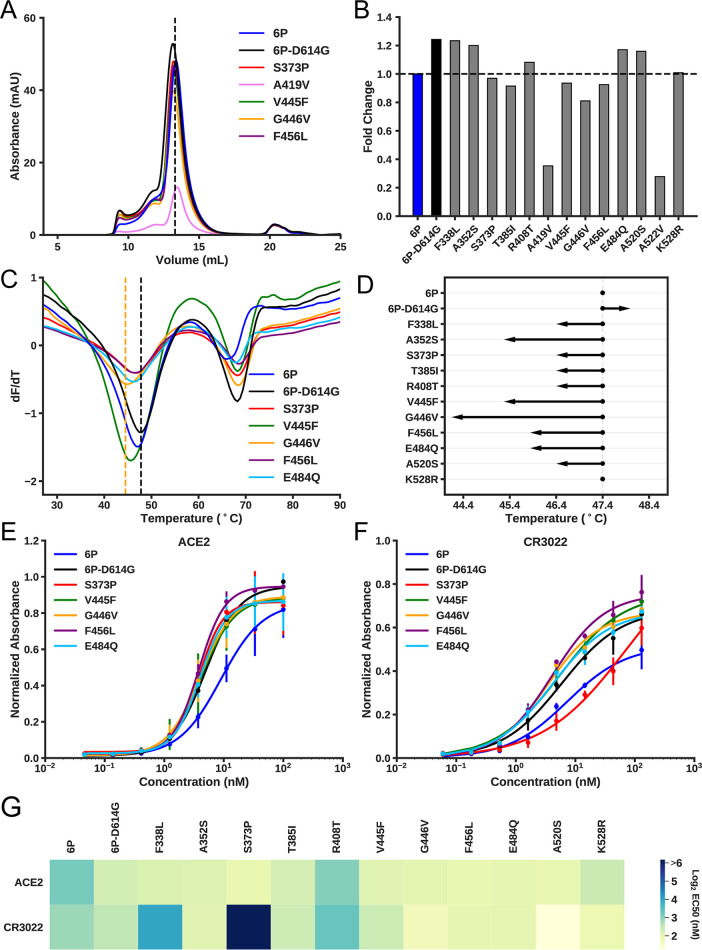
Biochemical characterization of spike RBD variants. (A) Size exclusion chromatography (SEC) traces of the indicated spike-RBD variants. The dashed line indicates the elution peak of spike-6P. mAU, milli-absorbance units. (B) Relative expression levels of all RBD variants as determined by the area under the SEC traces. All expression levels are normalized relative to spike-6P. (C) Thermostability analysis of RBD variants by differential scanning fluorimetry. Each sample had three replicates, and only mean values were plotted. The black vertical dashed line indicates the first melting temperature of 6P-D614G, and the orange vertical dashed line indicates the first melting temperature of the least stable variant (spike-G446V). (D) First apparent melting temperatures of all RBD variants. (E and F) ELISA-based binding affinities for ACE2 receptor (E) and the neutralizing antibody CR3022 (F) to the indicated RBD variants. (G) Summary of EC_50_ values for all measured RBD variants.

The RBD of spike protein binds the ACE2 surface receptor and is also targeted by neutralizing antibodies (35, 36, 40, 42–45, 47–61, 70). Thus, single amino acid replacements in this domain may have functional consequences that enhance virus fitness. To begin to test this idea, we expressed spike variants with the Asp614Gly replacement and 13 clinical RBD variants identified in our genome sequencing studies (Fig. 8; see also Table S4A and B). All RBD variants were cloned into an engineered spike protein construct that stabilizes the perfusion state and increases overall expression yield (spike-6P, here referred to as spike) (63).

10.1128/mBio.02707-20.10 TABLE S4 Primers and plasmids used for the *in vitro* characterization of recombinant proteins with single amino acid replacements in the receptor binding domain (RBD) region of spike protein and their biophysical properties. To test the hypothesis that RBD amino acid changes enhance viral fitness, we expressed spike variants with the Asp614Gly replacement and 13 clinical RBD variants identified in our genome sequencing studies. Panel A lists the primers used, panel B lists the plasmid construct information, and panel C lists the biophysical properties of the resultant spike protein variants. Download Table S4, PDF file, 0.1 MB. Copyright © 2020 Long et al. 2020 Long et al. This content is distributed under the terms of the Creative Commons Attribution 4.0 International license.

We first assessed the biophysical properties of spike-Asp614Gly, an amino acid polymorphism that is common globally and that was present at significantly increased levels in our wave 2 strain isolates. Pseudotyped viruses expressing spike-Gly614 have higher infectivity for host cells *in vitro* than spike-Asp614 (65, 66, 68, 71, 72). The higher infectivity of spike-Gly614 is correlated with increased stability and incorporation of the spike protein into the pseudovirion (72). We observed a higher expression level (Fig. 8A and B) and increased thermostability for the spike protein construct containing this variant (Fig. 8C and D). The size exclusion chromatography (SEC) elution profile of spike-Asp614 was indistinguishable from that of spike-Gly614, consistent with a trimeric conformation (Fig. 8A). These results are broadly consistent with higher-resolution structural analyses of both spike variants.

Next, we purified and biophysically characterized 13 RBD mutants that each contain Gly614 and one additional single amino acid replacement that we identified by genome sequencing of our clinical samples (Table S4C). All variants eluted as trimers, indicating that the global structure remained intact (Fig. 8; see also Fig. S6). However, several variants had reduced expression levels and virtually all had decreased thermostability relative to the variant that had only a single D614G amino acid replacement (Fig. 8D). The A419V and A522V mutations were especially deleterious, reducing yield and precluding further downstream analysis (Fig. 8B). We next assayed the affinity of the 11 highest-expressing spike variants for ACE2 receptor and the neutralizing monoclonal antibody CR3022 via enzyme-linked immunosorbent assays (ELISAs) (Fig. 8E to G; see also Table S4C). Most variants retained high affinity for the ACE2 surface receptor. However, importantly, three RBD variants (F338L, S373P, and R408T) had substantially reduced affinity for CR3022, a monoclonal antibody that disrupts the spike protein homotrimerization interface (62, 73). Notably, the S373P mutation is one amino acid away from the epitope recognized by CR3022 (62). These results are consistent with the interpretation that some RBD mutants arising in COVID-19 patients may have an increased ability to escape humoral immune pressure but otherwise retain strong ACE2 binding affinity.

10.1128/mBio.02707-20.6 FIG S6 Biochemical characterization of single amino acid variants of spike protein RBD. (A and B) Size exclusion chromatography (SEC) traces of the indicated spike-RBD variants. The dashed line indicates the elution peak of spike-6P. (C) Thermostability analysis of RBD variants. Each sample had three replicates, and only mean values were plotted. The black vertical dashed line indicates the first melting temperature of 6P-D614G. (D and E) ELISA-based binding affinities of ACE2 (D) and neutralizing monoclonal antibody CR3022 (E) to the indicated RBD variants. Download FIG S6, PDF file, 0.3 MB. Copyright © 2020 Long et al. 2020 Long et al. This content is distributed under the terms of the Creative Commons Attribution 4.0 International license.

## DISCUSSION

In this work, we analyzed the molecular population genomics, sociodemographic, and medical features of two waves of COVID-19 disease occurring in metropolitan Houston, TX, between early March and early July 2020. We also studied the biophysical and immunologic properties of some naturally occurring single amino acid changes in the spike protein RBD identified by sequencing the 5,085 genomes. We discovered that the first COVID-19 wave was caused by a heterogenous array of virus genotypes assigned to several different clades. The majority of cases in the first wave were related to strains that caused widespread disease in European and Asian countries, as well as other localities. We conclude that the SARS-CoV-2 virus was introduced into Houston many times independently, likely by individuals who had traveled to or from different parts of the world, including other communities in the United States. In support of this conclusion, the first cases in metropolitan Houston were associated with a travel history to a region with a known high incidence of COVID-19 (15). The data are consistent with the fact that Houston is a large international city characterized by a multiethnic population and is a prominent transport hub with direct flights to major cities globally.

The second wave of COVID-19 cases is also characterized by SARS-CoV-2 strains with diverse genotypes. Virtually all cases in the second and ongoing disease wave had been caused by strains with the Gly614 variant of spike protein (Fig. 1B). Our data unambiguously demonstrate that strains with the Gly614 variant increased significantly in frequency in wave 2 relative to wave 1 in the Houston metropolitan region. This shift occurred very rapidly, in a matter of just a few months. Amino acid residue Asp614 is located in subdomain 2 (SD-2) of the spike protein and forms a hydrogen bond and electrostatic interaction with two residues in the S2 subunit of a neighboring protomer. Replacement of aspartate with glycine would eliminate both interactions, thereby substantively weakening the contact between the S1 and S2 subunits. We previously speculated (74) that this weakening produces a more highly fusogenic spike protein, as S1 must first dissociate from S2 before S2 can refold and mediate fusion of virus and cell membranes. Stated another way, virus strains with the Gly614 variant may be better able to enter host cells, potentially resulting in enhanced spread. Consistent with this idea, Korber et al. (65) showed that the Gly614 variant grows to a higher titer as pseudotyped virions. On initial diagnosis, infected individuals had lower real-time PCR (RT-PCR) cycle threshold values, suggesting higher upper respiratory tract viral loads. Our data (Fig. 7) are fully consistent with the finding, previously reported by Zhang et al. (72), that pseudovirus with the 614Gly variant infected ACE2 receptor-expressing cells more efficiently than the 614Asp variant. Similar results have been described by Hu et al. (66) and Lorenzo-Redondo et al. (67). Plante et al. (75) recently studied isogenic mutant SARS-CoV-2 strains with either the 614Asp or 614Gly variant and found that the 614Gly variant virus showed significantly increased replication in human lung epithelial cells *in vitro* and increased infectious titers in nasal and tracheal washes obtained from experimentally infected hamsters. These results are consistent with the idea that the 614Gly variant bestows increased virus fitness in the upper respiratory tract (75).

Additional work is needed to investigate the potential biomedical relevance and public health importance of the Asp614Gly polymorphism, including but not limited to virus dissemination, overall fitness, impact on clinical course and virulence, and development of vaccines and therapeutics. Although it is possible that stochastic processes alone may account for the rapid increase in COVID-19 disease frequency caused by viruses containing the Gly614 variant, we do not favor that interpretation, in part because of the cumulative weight of the epidemiologic, human RT-PCR diagnostics data, *in vitro* experimental findings, and animal infection studies using isogenic mutant virus strains (65–69, 72, 75). In addition, if stochastic processes are solely responsible, we believe it is difficult to explain essentially simultaneous increases in frequency of the Gly614 variant in genetically diverse viruses in three distinct clades (G, GH, and GR) in a geographically large metropolitan area with 7 million ethnically diverse people. Regardless, more research on this important topic is warranted.

The diversity present in our 1,026 virus genomes from the first disease wave contrasts somewhat with data reported by Gonzalez-Reiche et al., who studied 84 SARS-CoV-2 isolates causing disease in patients in the New York City region (11). Those investigators concluded that the vast majority of disease was caused by progeny of strains imported from Europe. Similarly, Bedford et al. (10) reported that much of the COVID-19 disease in the Seattle, WA, area was caused by strains that are progeny of a virus strain recently introduced from China. Some aspects of our findings are similar to those reported recently by Lemieux et al. on the basis of analysis of strains causing disease in the Boston area (76). Our findings, like theirs, highlight the importance of multiple importation events of genetically diverse strains in the epidemiology of COVID-19 disease in this pandemic. Similarly, Icelandic and Brazilian investigators documented that SARS-CoV-2 was imported by individuals traveling to or from many European and other countries (77, 78).

The virus genome diversity and large sample size in our study permitted us to test the hypothesis that distinct virus clades were nonrandomly associated with hospitalized COVID-19 patients or disease severity. We did not find evidence to support this hypothesis, but our continuing study of COVID-19 cases accruing in the second wave will further improve statistical stratification.

We used machine learning classifiers to identify if any SNPs contribute to increased infection severity or otherwise affect virus-host outcomes. The models could not be trained to accurately predict these outcomes from the available virus genome sequence data. This may have been due to sample size or class imbalance. However, we do not favor this interpretation. Rather, we think that the inability to identify particular virus SNPs predictive of disease severity or infection outcome likely reflects the substantial heterogeneity in underlying medical conditions and treatment regimens of the COVID-19 patients studied here. An alternative but not mutually exclusive hypothesis is that patient genotypes play an important role in determining virus-human interactions and in the resulting pathology. Although some evidence has been presented in support of this idea (79, 80), available data suggest that in the aggregate, host genetics does not play an overwhelming role in determining outcome in the great majority of adult patients, once virus infection is established.

Remdesivir is a nucleoside analog reported to have activity against MERS-CoV, a coronavirus related to SARS-CoV-2. Recently, several studies have reported that remdesivir shows promise in treating COVID-19 patients (28–32), leading the FDA to issue an emergency use authorization. Because *in vitro* resistance of SARS-CoV to remdesivir has been reported to be caused by either of two amino acid replacements in RdRp (Phe479Leu or Val556Leu), we interrogated our data for polymorphisms in the *nsp12* gene. Although we identified 140 different inferred amino acid replacements in RdRp in the 5,085 genomes analyzed, none of these were located precisely at the two positions associated with *in vitro* resistance to remdesivir. Inasmuch as remdesivir is now being deployed widely to treat COVID-19 patients in Houston and elsewhere, our findings suggest that the majority of SARS-CoV-2 strains currently circulating in our region should be susceptible to this drug.

The amino acid replacements Ala442Val, Ala448Val, Ala553Pro/Val, and Gly682Arg that we identified occur at sites that, intriguingly, are located directly above the nucleotide substrate entry channel and nucleotide binding residues Lys544, Arg552, and Arg554 (21, 22) (Fig. 4). One possibility is that substitution of the smaller alanine or glycine residues with the bulkier side chains of Val/Pro/Arg may impose structural constraints for the modified nucleotide analog to bind and may thereby disfavor remdesivir binding. This, in turn, may lead to reduced incorporation of remdesivir into the nascent RNA, increased fidelity of RNA synthesis, and, ultimately, drug resistance. A similar mechanism has been proposed for a Val556Leu change (22).

We also identified one strain with a Lys477Asn replacement in RdRp. This substitution is located close to a Phe479Leu replacement reported to have produced partial resistance to remdesivir *in vitro* in SARS-CoV patients from 2004, although the amino acid positions are numbered differently in SARS-CoV and SARS-CoV-2. Structural studies have suggested that this amino acid is surface exposed and is distant from known key functional elements. Our observed Lys477Asn change is also located in a conserved motif described as a finger domain of RdRp (Fig. 3 and 4). One speculative possibility is that Lys477 is involved in binding an as-yet-unidentified cofactor such as Nsp7 or Nsp8, an interaction that could modify nucleotide binding and/or fidelity at a distance. These data warrant additional study in larger patient cohorts, especially in individuals treated with remdesivir.

Analysis of the gene encoding the spike protein identified 285 polymorphic amino acid sites relative to the reference genome, including 49 inferred amino acid replacements not present in available databases as of 19 August 2020. Importantly, 30 amino acid sites in the spike protein had two or three distinct replacements relative to the reference strain. The occurrence of multiple variants at the same amino acid site is one characteristic that may suggest functional consequences. These data, coupled with structural information available for spike protein, raise the possibility that some of the amino acid variants have functional consequences, including, for example, altered serologic reactivity as shown here. These data permit generation of many biomedically relevant hypotheses now under study.

A recent study reported that RBD amino acid changes could be selected *in vitro* using a pseudovirus neutralization assay and sera obtained from convalescent plasma or monoclonal antibodies (81). The amino acid sites included positions V445 and E484 in the RBD. Note that variants G446V and E484Q were present in our patient samples. However, these mutations retain high affinity to CR3022 (Fig. 8F and G). The high-resolution structure of the RBD/CR3022 complex shows that CR3022 makes contacts to residues 369 to 386, 380 to 392, and 427 to 430 of RBD (73). Although there is no overlap of CR3022 and ACE2 receptor epitopes, CR3022 is able to neutralize the virus through an allosteric effect. We found that the Ser373Pro change, which is located within the CR3022 epitope, resulted in reduced affinity to CR3022 (Fig. 8F and G). The F338L and R408T mutations, although not found directly within the interacting epitope, also display reduced binding to CR3022. Other investigators (81) using *in vitro* antibody selection identified a change at amino acid site S151 in the N-terminal domain, and we found mutations S151N and S151I in our patient samples. We also note that two variant amino acids (Gly446Val and Phe456Leu) that we identified were located in a linear epitope found to be critical for a neutralizing monoclonal antibody described recently by Li et al. (82).

In the aggregate, these findings suggest that mutations emerging within the spike protein at positions within and proximal to known neutralization epitopes may result in escape from antibodies and other therapeutics currently under development. Importantly, our study did not reveal that these mutant strains had disproportionately increased in number over time. The findings may also bear on the occurrence of multiple amino acid substitutions at the same amino acid site that we identified in this study, commonly a signal of selection. In the aggregate, the data support a multifaceted approach to serological monitoring and biologics development, including the use of monoclonal antibody cocktails (45, 46, 83).

### Concluding statement.

Our work represents analysis of the largest sample to date of SARS-CoV-2 genome sequences from patients in one metropolitan region in the United States. The investigation was facilitated by the fact that we had rapidly assessed a SARS-CoV-2 molecular diagnostic test in January 2020, more than a month before the first COVID-19 patient was diagnosed in Houston. In addition, our large health care system has seven hospitals and many facilities (e.g., outpatient care centers, emergency departments) located in geographically diverse areas of the city. We also provide reference laboratory services for other health care entities in the Houston area. Together, our facilities serve patients of diverse ethnicities and socioeconomic statuses. Thus, the data presented here likely reflect a broad overview of virus diversity causing COVID-19 infections throughout metropolitan Houston. We previously exploited these features to study influenza virus and Klebsiella pneumoniae dissemination in metropolitan Houston (84, 85). We acknowledge that not every “twig” of the SARS-CoV-2 evolutionary tree in Houston is represented in these data. The samples studied are not comprehensive with respect to the entire metropolitan region. For example, it is possible that our strain samples are not fully representative of individuals who are indigent, homeless, or of very low socioeconomic status. In addition, although the strain sample size was relatively large compared to other studies, the samples represented only about 10% of all COVID-19 cases in metropolitan Houston documented in the study period. In addition, some patient samples contained relatively small amounts of virus nucleic acid and did not yield adequate sequence data for high-quality genome analysis. Thus, our data likely underestimate the extent of genome diversity present among SARS-CoV-2 strains causing COVID-19 and will not identify all amino acid replacements in the virus in this geographic region. It will be important to sequence and analyze the genomes of additional SARS-CoV-2 strains causing COVID-19 cases in the ongoing second massive disease wave in metropolitan Houston, and such studies are under way. Data of this type will be especially important to have if a third wave and subsequent waves were to occur in metropolitan Houston, as it could provide insight into molecular and epidemiologic events contributing to them.

The genomes reported here are an important data resource that will underpin our ongoing study of SARS-CoV-2 molecular evolution and dissemination and medical features of COVID-19 in Houston. As of 19 August 2020, there were 135,866 reported cases of COVID-19 in metropolitan Houston, and the number of cases is increasing daily. Although the full array of factors contributing to the massive second wave in Houston is not known, it is possible that the potential for increased transmissibility of SARS-CoV-2 with the Gly614 amino acid replacement may have played a role, as well as changes in behavior associated with the Memorial Day and July 4th holidays and relaxation of some of the social constraints imposed during the first wave. The availability of extensive virus genome data dating from the earliest reported cases of COVID-19 in metropolitan Houston, coupled with the database we have now constructed, may provide critical insights into the origin of the new infection spikes and waves that are occurring as public health constraints are further relaxed, schools and colleges reopen, holidays occur, commercial air travel increases, and individuals change their behavior because of COVID-19 “fatigue.” The genome data will also be useful in assessing ongoing molecular evolution in spike and other proteins as baseline herd immunity is generated, either by natural exposure to SARS-CoV-2 or by vaccination. The signal of potential selection contributing to some spike protein diversity and identification of naturally occurring mutant RBD variants with altered serologic recognition warrant close attention and expanded study.

## MATERIALS AND METHODS

### Patient specimens.

All specimens were obtained from individuals who were registered patients at Houston Methodist hospitals, associated facilities (e.g., urgent care centers), or institutions in the greater Houston metropolitan region that use our laboratory services. Virtually all individuals met the criteria specified by the Centers for Disease Control and Prevention to be classified as a person under investigation.

### SARS-CoV-2 molecular diagnostic testing.

Specimens obtained from symptomatic patients with a high degree of suspicion for COVID-19 disease were tested in the Molecular Diagnostics Laboratory at Houston Methodist Hospital using an assay granted Emergency Use Authorization (EUA) from the FDA (https://www.fda.gov/medical-devices/emergency-situations-medical-devices/faqs-diagnostic-testing-sars-cov-2#offeringtests). Multiple testing platforms were used, including an assay that follows the protocol published by the WHO (https://www.who.int/docs/default-source/coronaviruse/protocol-v2-1.pdf) using an EZ1 virus extraction kit and an EZ1 Advanced XL instrument or a QIASymphony DSP virus kit and a QIASymphony instrument for nucleic acid extraction and an ABI 7500 Fast Dx instrument with 7500 SDS software for reverse transcription RT-PCR, the COVID-19 test using BioFire Film Array 2.0 instruments, the Xpert Xpress SARS-CoV-2 test using Cepheid GeneXpert Infinity or Cepheid GeneXpert Xpress IV instruments, the SARS-CoV-2 assay using a Hologic Panther instrument, and the Aptima SARS-CoV-2 assay using a Hologic Panther Fusion system. All assays were performed according to the manufacturer’s instructions. Testing was performed on material obtained from nasopharyngeal or oropharyngeal swabs immersed in universal transport media (UTM), bronchoalveolar lavage fluid, or sputum treated with dithiothreitol (DTT). To standardize specimen collection, an instructional video was created for Houston Methodist Hospital health care workers (https://vimeo.com/396996468/2228335d56).

### Epidemiologic curve.

The number of confirmed COVID-19-positive cases was obtained from USAFacts.org (https://usafacts.org/visualizations/coronavirus-covid-19-spread-map/) for Austin, Brazoria, Chambers, Fort Bend, Galveston, Harris, Liberty, Montgomery, and Waller counties. COVID-19-positive cases for Houston Methodist Hospital patients were obtained from our Laboratory Information System and plotted using the documented collection time.

### SARS-CoV-2 genome sequencing.

Libraries for whole-virus genome sequencing were prepared according to version 1 or version 3 of the ARTIC nCoV-2019 sequencing protocol (https://artic.network/ncov-2019). Long reads were generated with the LSK-109 sequencing kit, 24 native barcodes (NBD104 and NBD114 kits), and a GridION instrument (Oxford Nanopore). Short reads were generated with a NexteraXT kit and a NextSeq 550 instrument (Illumina).

### SARS-CoV-2 genome sequence analysis.

Consensus virus genome sequences from the Houston area isolates were generated using the ARTIC nCoV-2019 bioinformatics pipeline. Publicly available genomes and metadata were acquired through GISAID on 19 August 2020. GISAID sequences containing greater than 1% N characters and Houston sequences with greater than 5% N characters were removed from consideration. Identical GISAID sequences originating from the same geographic location with the same collection date were also removed from consideration to reduce redundancy. Nucleotide sequence alignments for the combined Houston and GISAID strains were generated using MAFFT version 7.130b with default parameters (86). Sequences were manually curated in JalView (87) to trim the ends and to remove sequences containing spurious inserts. Phylogenetic trees were generated using FastTree with the generalized time-reversible model for nucleotide sequences (88). CLC Genomics Workbench (Qiagen) was used to generate the phylogenetic tree figures.

### Geospatial mapping.

The home address Zip code for all SARS-CoV-2-positive patients was used to generate the geospatial maps. To examine geographic relatedness among genetically similar isolates, geospatial maps were filtered for isolates containing specific amino acid changes.

### Time series.

Geospatial data were filtered into wave 1 (5 March 2020 to 11 May 2020) and wave 2 (12 May 2020 to 7 July 2020) time intervals to illustrate the spread of confirmed SARS-CoV-2-positive patients identified over time.

### Machine learning.

Virus genome alignments and patient metadata were used to build models to predict patient metadata and outcomes using both classification models and regression. Metadata considered for prediction in the classification models included age, ABO and Rh blood type, ethnic group, ethnicity, sex, ICU admission, IMU admission, supplemental oxygen use, and ventilator use. Metadata considered for prediction in regression analysis included ICU length of stay, IMU length of stay, total length of stay, supplemental oxygen use, and ventilator use. Because sex, blood type, Rh factor, age, age decade, ethnicity, and ethnic group are features in the patient features and combined feature sets, models were not trained for these labels using patient and combined feature sets. Additionally, age, length of stay, IMU length of stay, ICU length of stay, mechanical ventilation days, and supplemental oxygen days were treated as regression problems and XGBoost regressors were built while the rest were treated as classification problems and XGBoost classifiers were built.

Three types of features were considered for training the XGBoost classifiers: alignment features, patient features, and the combination of alignment and patient features. Alignment features were generated from the consensus genome alignment such that columns containing ambiguous nucleotide bases were removed to ensure that the models did not learn patterns from areas of low coverage. These alignments were then one-hot encoded to form the alignment features. Patient metadata values were one-hot encoded with the exception of age, which remained as a raw integer value, to create the patient features. These metadata values consisted of age, ABO, Rh blood type, ethnic group, ethnicity, and sex. All three types of feature sets were used to train models that predict ICU length of stay, IMU length of stay, overall length of stay, days of supplemental oxygen therapy, and days of ventilator usage, while only alignment features were used to train models that predict age, ABO, Rh blood type, ethnic group, ethnicity, and sex.

A 10-fold cross validation was used to train XGBoost models (89) as described previously (90, 91). Depths of 4, 8, 16, 32, and 64 were used to tune the models, but the accuracies plateaued after a depth of 16. SciKit-Learn’s (92) classification report and *R*^2^ score were then used to access the overall accuracy of the classification and regression models, respectively.

### Patient metadata correlations.

We encoded values into multiple columns for each metadata field for patients if metadata was available. For example, the ABO column was divided into four columns for A, B, AB, and O blood type. Those columns were encoded with a 1 for the patients’ ABO type, with all other columns encoded with 0. This was repeated for all nonoutcome metadata fields. Age, however, was not reencoded, as the raw integer values were used. Each column was then correlated to the various outcome values for each patient (deceased, ICU length, IMU length, length of stay, supplemental oxygen length, and ventilator length) to obtain a Pearson coefficient correlation value for each metadata label and outcome.

### Analysis of the *nsp12* polymerase and S protein genes.

The *nsp12* virus polymerase and S protein genes were analyzed by plotting SNP density in the consensus alignment using Python (Python v3.4.3, Biopython Package v1.72). The frequency of SNPs in the Houston isolates was assessed, along with amino acid changes for nonsynonymous SNPs.

### Cycle threshold (*C_T_*) comparison of SARS-CoV-2 strains with either Asp614 or Gly614 amino acid replacements in the spike protein.

The cycle threshold (*C_T_*) value for every sequenced strain that was detected from a patient specimen using the SARS-CoV-2 assay on a Hologic Panther instrument was retrieved from the Houston Methodist Hospital Laboratory Information System. The statistical significance of results of comparisons between the mean *C_T_* values for strains with an aspartate (*n *= 102) or glycine (*n *= 812) amino acid at position 614 of the spike protein was determined with the Mann-Whitney test (GraphPad Prism 8).

### Creation and characterization of spike protein RBD variants.

Spike RBD variants were cloned into the spike-6P (HexaPro; F817P, A892P, A899P, A942P, K986P, V987P) base construct that also includes the D614G substitution (pIF638). Briefly, a segment of the gene encoding the RBD was excised with EcoRI and NheI, mutagenized by PCR, and assembled with a HiFi DNA assembly cloning kit (NEB).

FreeStyle 293-F cells (Thermo Fisher Scientific) were cultured and maintained in a humidified atmosphere of 37°C and 8% CO_2_ with shaking at 110 to 125 rpm. Cells were transfected with plasmids encoding spike protein variants using polyethylenimine. Three hours posttransfection, 5 μM kifunensine was added to each culture. Cells were harvested 4 days after transfection, and the protein-containing supernatant was separated from the cells by two centrifugation steps: 10 min at 500 relative centrifugal force (rcf) and 20 min at 10,000 rcf. Supernatants were kept at 4°C throughout. Clarified supernatant was loaded on a Poly-Prep chromatography column (Bio-Rad) containing Strep-Tactin Superflow resin (IBA), washed with five column volumes (CV) of wash buffer (100 mM Tris-HCl [pH 8.0], 150 mM NaCl, 1 mM EDTA), and eluted with four CV of elution buffer (100 mM Tris-HCl [pH 8.0], 150 mM NaCl, 1 mM EDTA, 2.5 mM d-desthiobiotin). The eluate was spin concentrated (Amicon Ultra-15) to 600 μl and further purified via size exclusion chromatography (SEC) using a Superose 6 Increase 10/300 column (GE) and SEC buffer (2 mM Tris [pH 8.0], 200 mM NaCl, 0.02% NaN_3_). Proteins were concentrated to 300 μl and stored in SEC buffer.

The RBD spike mutants chosen for analysis were all RBD amino acid mutants identified by our genome sequencing study as of 15 June 2020. We note that the exact boundaries of the RBD domain vary depending on the paper used as the reference. We used the boundaries demarcated in Fig. 1A of the article by Cai et al. [*Science*, 21 July]) (93) that have K528R located at the RBD-CTD1 interface.

### Differential scanning fluorimetry.

Recombinant spike proteins were diluted to a final concentration of 0.05 mg/ml with 5× SYPRO orange (Sigma) in a 96-well qPCR plate. Continuous fluorescence measurements (λ excitation [λex] = 465 nm, λ emission [λem] = 580 nm) were collected with a Roche LightCycler 480 II instrument. The temperature was increased from 22°C to 95°C at a rate of 4.4°C/min. We report the first melting transition.

### Enzyme-linked immunosorbent assays.

ELISAs were performed to characterize binding of S6P, S6P D614G, and S6P D614G-RBD variants to human ACE2 and the RBD-binding monoclonal antibody CR3022. The ACE2-hFc chimera was obtained from GenScript (Z03484), and the CR3022 antibody was purchased from Abcam (Ab273073). Corning 96-well high-binding plates (CLS9018BC) were coated with spike variants at 2 μg/ml overnight at 4°C. After four washes with phosphate-buffered saline–0.1% Tween 20 (PBST; 300 μl/well), plates were blocked with PBS–2% milk (PBSM) for 2 h at room temperature and again washed four times with PBST. These were serially diluted in PBSM 1:3 seven times in triplicate. After 1 h of incubation at room temperature, plates were washed four times in PBST, labeled with 50 μl mouse anti-human IgG1 Fc-HRP (SouthernBiotech, 9054-05) for 45 min in PBSM, and washed again in PBST before addition of 50 μl 1-step Ultra TMB-ELISA substrate (Thermo Scientific, 34028). Reactions were developed for 15 min and stopped by addition of 50 μl 4 M H_2_SO_4_. Absorbance intensity (450 nm) was normalized within a plate, and 50% effective concentration (EC_50_) values were calculated through 4-parameter logistic curve (4PL) analysis using GraphPad Prism 8.4.3.
