# Supplementary material for: Molecular Architecture of Early Dissemination and Massive Second Wave of the SARS-CoV-2 Virus in a Major Metropolitan Area
Source: mBio. 2020 Oct 30;11(6):e02707-20. doi: 10.1128/mBio.02707-20 (PMC7642679; doi:10.1128/mBio.02707-20)
Supplement: TABLE S1 [file mBio.02707-20-st001.pdf]

Supplemental Table 1

	Wave 1, count (%)	Wave 2, count (%)
Number of patients with sequencing data	844	3839
Age (years)		
0-19	10 (1.2%)	168 (4.4%)
20-39	187 (22.2%)	1375 (35.8%)
40-59	352 (41.7%)	1316 (34.3%)
60-79	233 (27.6%)	819 (21.3%)
80-99	62 (7.3%)	161 (4.2%)
Average	53	46
Comorbidities		
Unknown	427 (50.6%)	2738 (71.3%)
Obesity-No	261 (62.6%)	725 (65.8%)
Obesity-Yes	156 (37.4%)	376 (34.2%)
Hypertension-No	183 (43.9%)	535 (48.6%)
Hypertension-Yes	234 (56.1%)	566 (51.4%)
Diabetes-No	238 (57.1%)	624 (56.7%)
Diabetes-Yes	179 (42.9%)	477 (43.3%)
COPD-No	333 (79.9%)	935 (84.9%)
COPD-Yes	84 (20.1%)	166 (15.1%)
CKD-No	327 (78.4%)	859 (78%)
CKD-Yes	90 (21.6%)	242 (22%)
Hyperlipidemia-No	250 (60%)	690 (62.7%)
Hyperlipidemia-Yes	167 (40%)	411 (37.3%)
Coronary Disease-No	347 (83.2%)	924 (83.9%)
Coronary Disease-Yes	70 (16.8%)	177 (16.1%)
Ethnic group		
Hispanic or Latino	215 (25.5%)	1408 (36.7%)
Not Hispanic or Latino	573 (67.9%)	2080 (54.2%)
Declined	11 (1.3%)	44 (1.1%)
Unavailable	45 (5.3%)	307 (8%)
Ethnicity		
Caucasian	404 (47.9%)	2146 (55.9%)

Black	275 (32.6%)	919 (23.9%)
Asian	83 (9.8%)	137 (3.6%)
Other	11 (1.3%)	114 (3.0%)
Unavailable	46 (5.5%)	318 (8.3%)
Declined	25 (3.0%)	205 (5.3%)
Median household income		
Number known	800 (94.8%)	3574 (93.1%)
Income	$68,204	$65,742
Mortality		
Living	750 (88.9%)	3466 (90.3%)
Deceased	56 (6.6%)	124 (3.2%)
Unavailable	38 (4.5%)	249 (6.5%)
Highest level of care		
ICU	186 (22%)	280 (7.3%)
IMU	7 (0.8%)	28 (0.7%)
Inpatient	224 (26.5%)	793 (20.7%)
Outpatient	389 (46.1%)	2489 (64.8%)
Unknown	38 (4.5%)	249 (6.5%)
