# Supplementary figures and images for: Molecular Architecture of Early Dissemination and Massive Second Wave of the SARS-CoV-2 Virus in a Major Metropolitan Area
Source: mBio. 2020 Oct 30;11(6):e02707-20. doi: 10.1128/mBio.02707-20 (PMC7642679; doi:10.1128/mBio.02707-20)

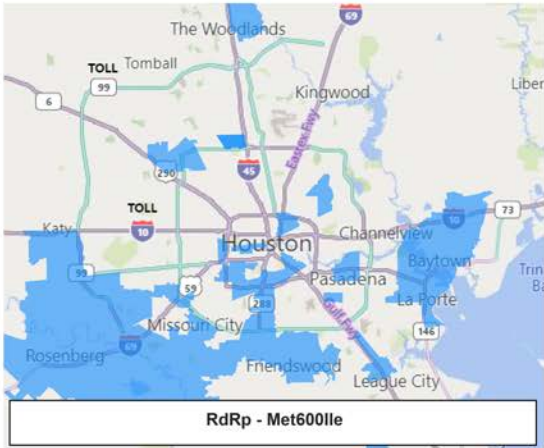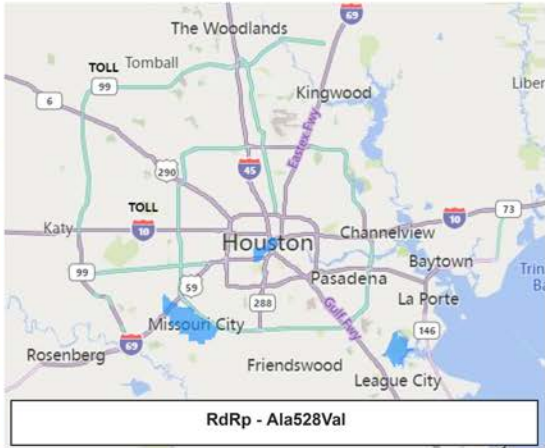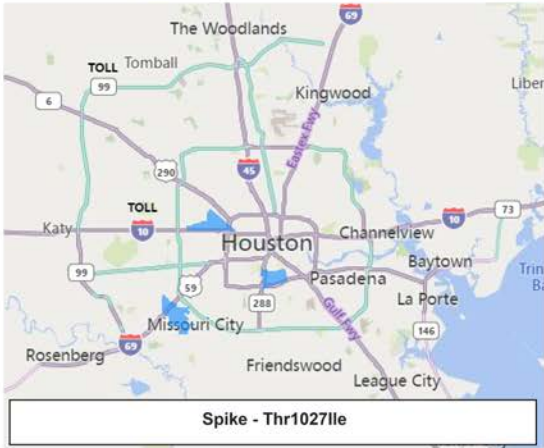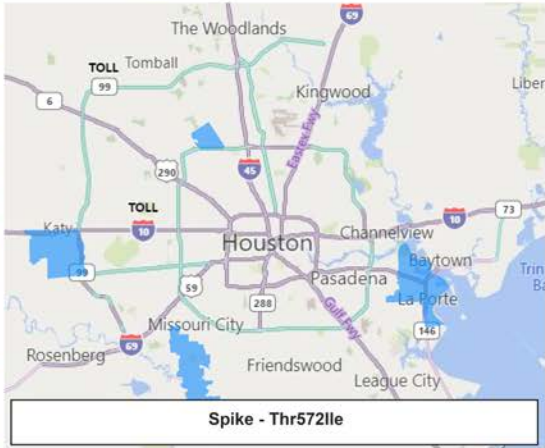

Supplement: FIG S1 [file mBio.02707-20-sf001.pdf]

A

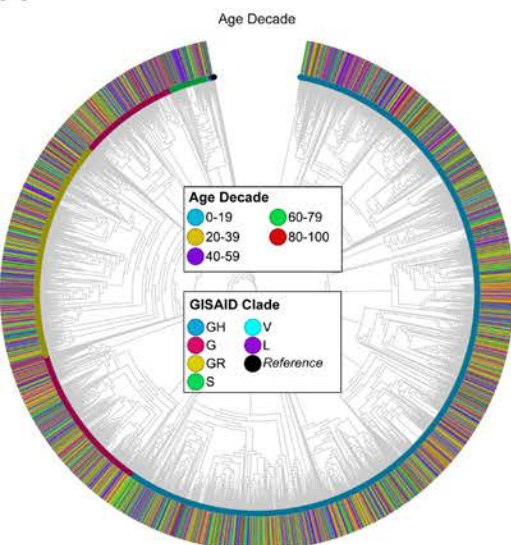

B

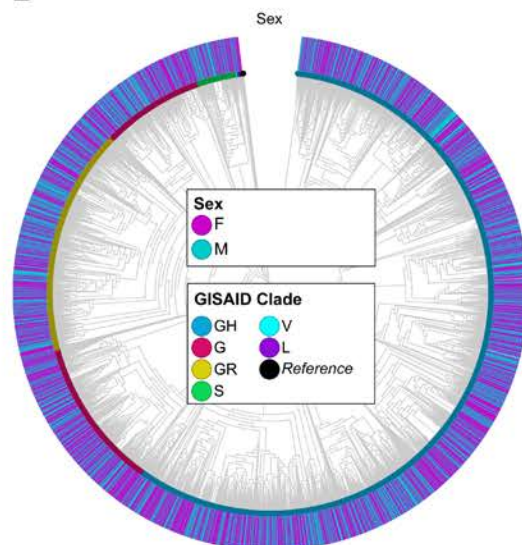

C

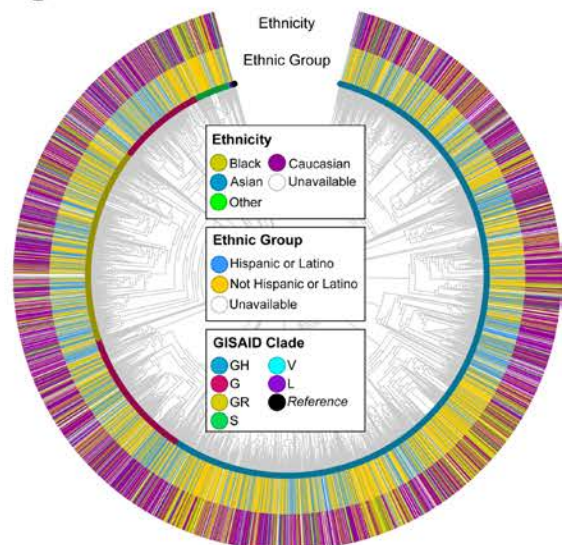

D

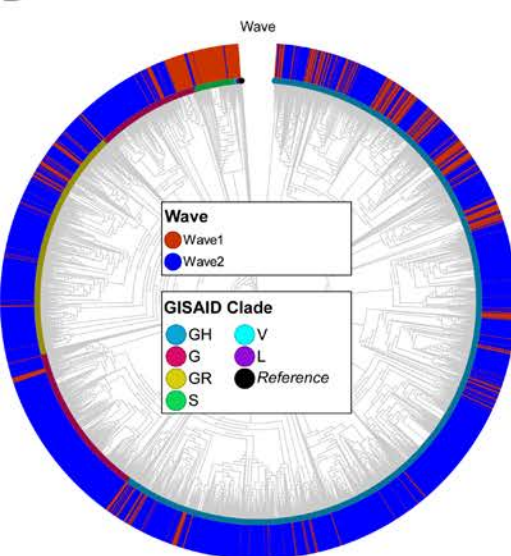

E

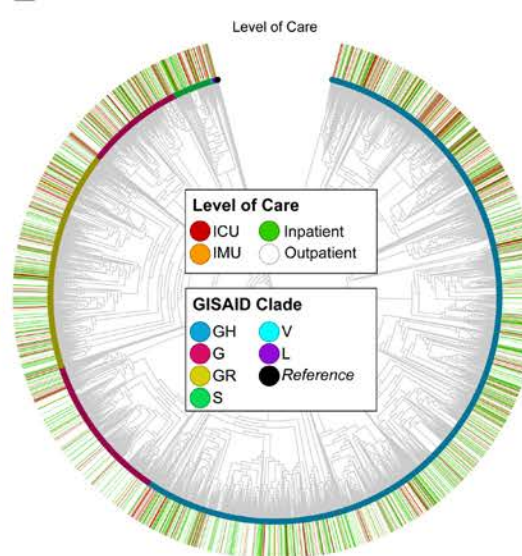

F

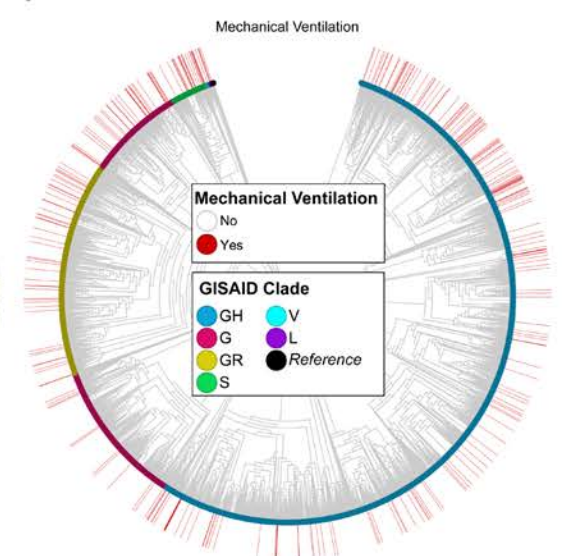

G

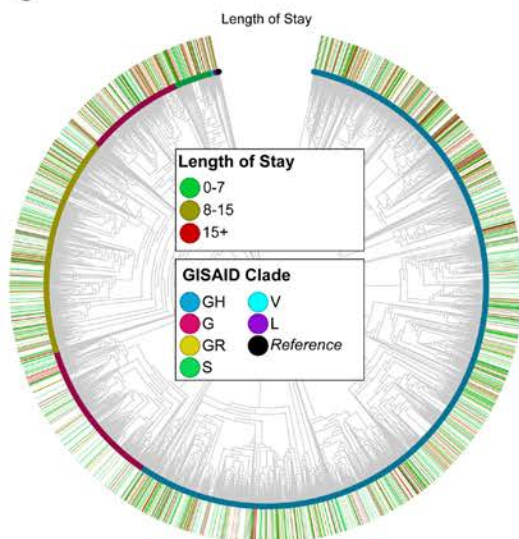

H

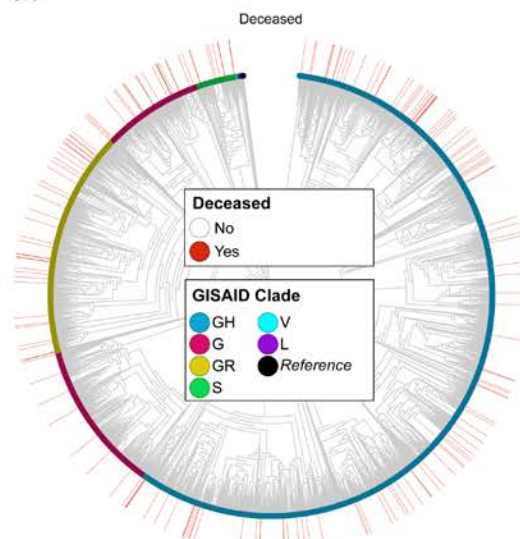

Supplement: FIG S2 [file mBio.02707-20-sf002.pdf]

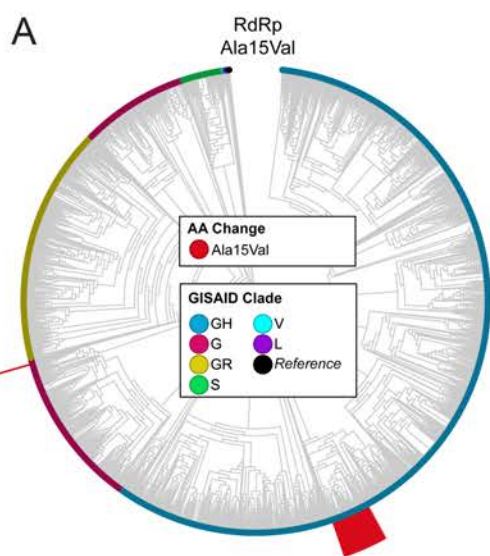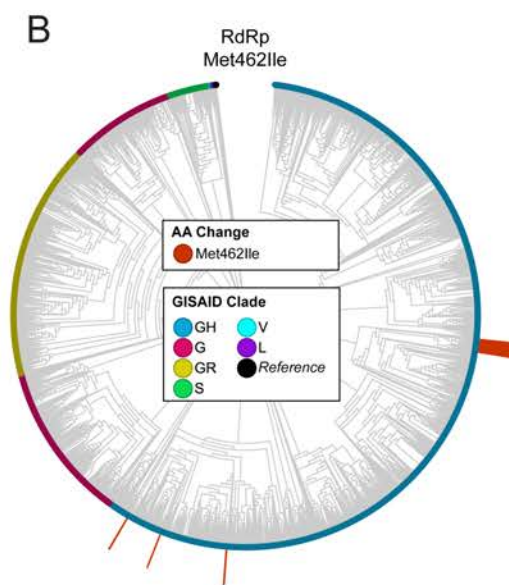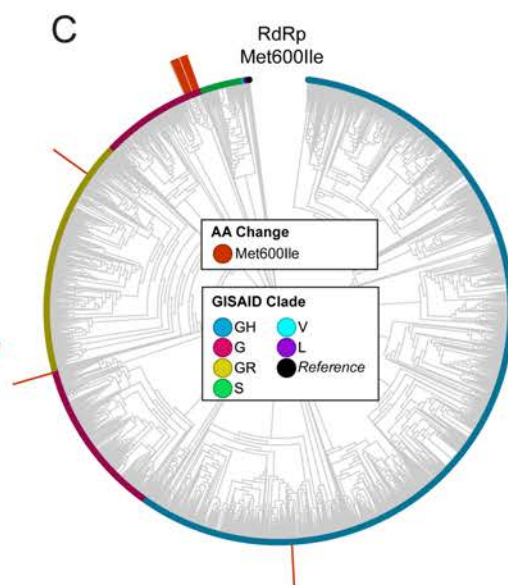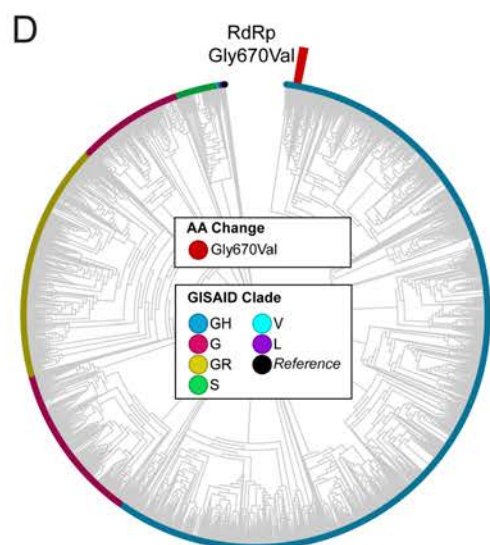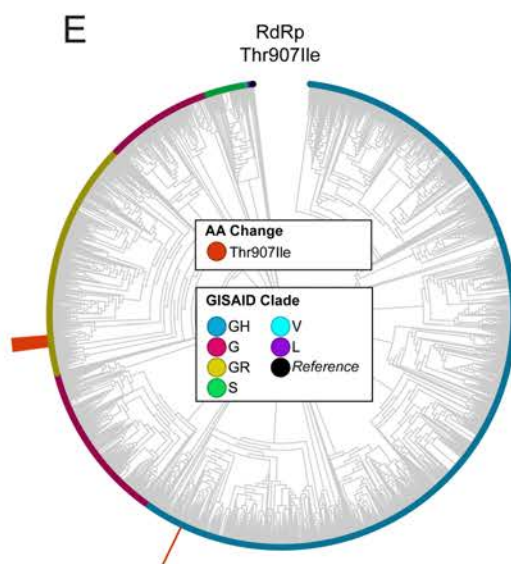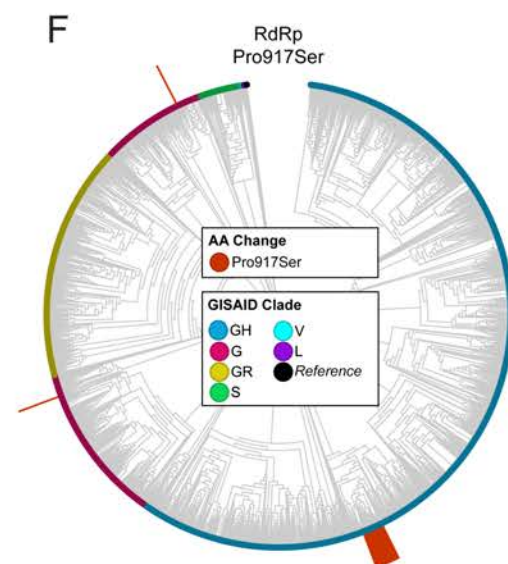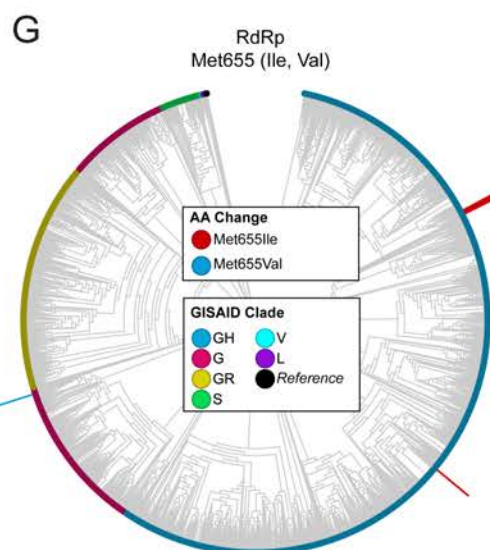

Supplement: FIG S3 [file mBio.02707-20-sf003.pdf]

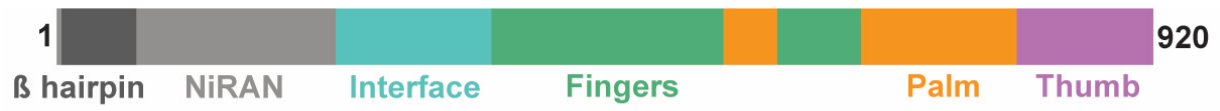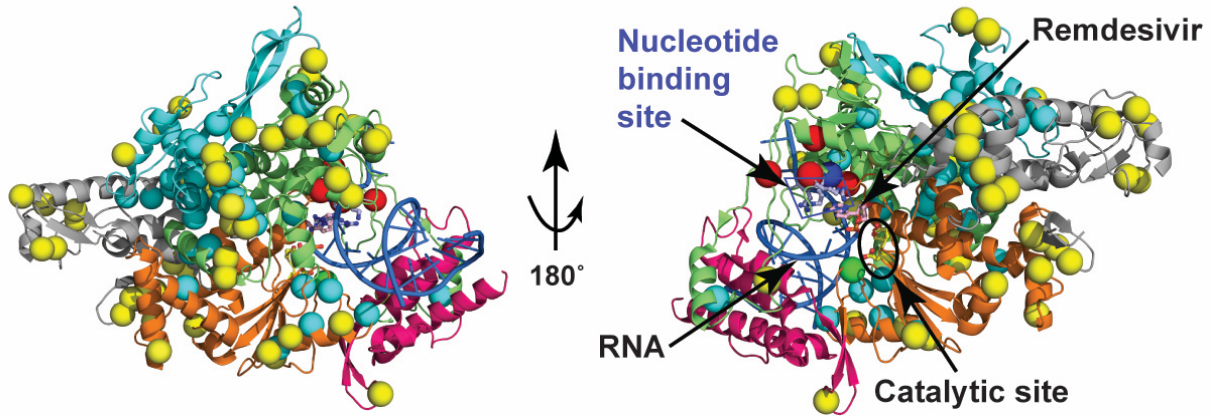

Supplement: FIG S4 [file mBio.02707-20-sf004.pdf]

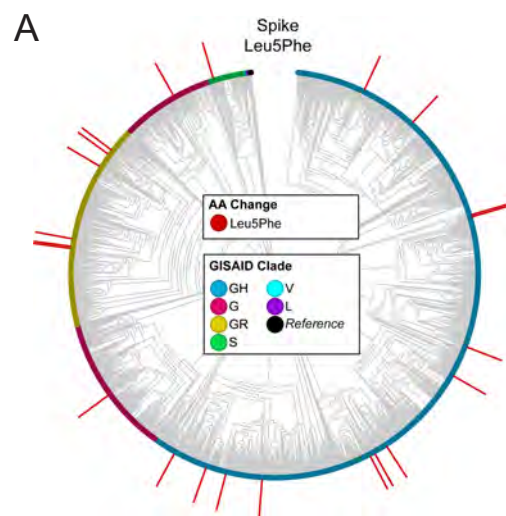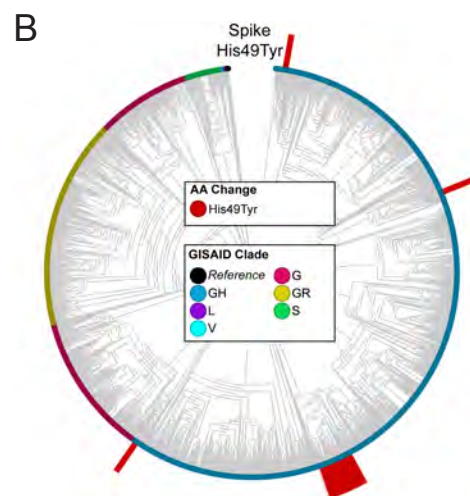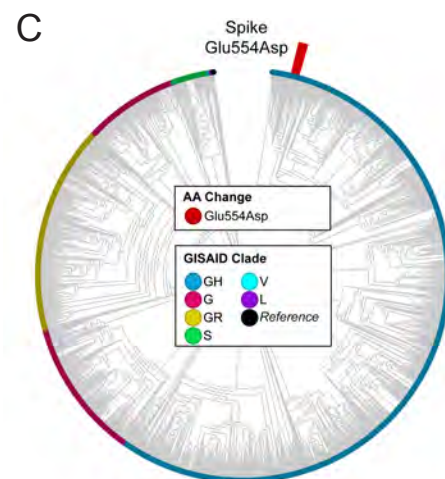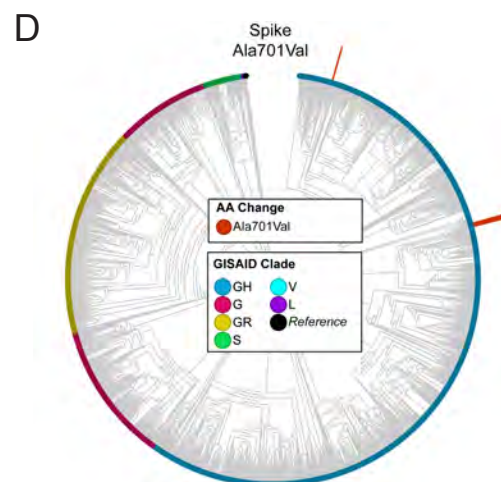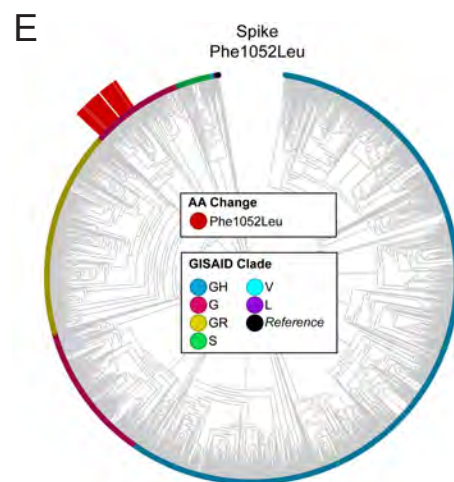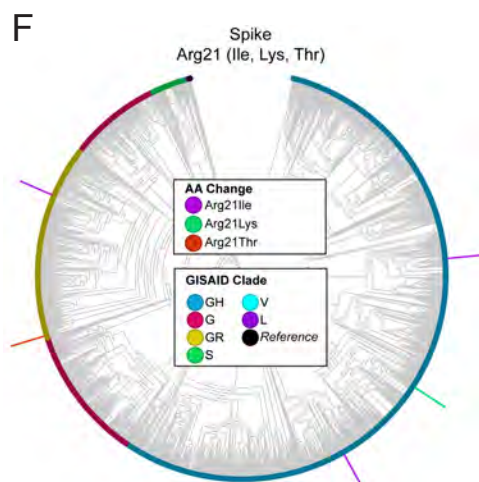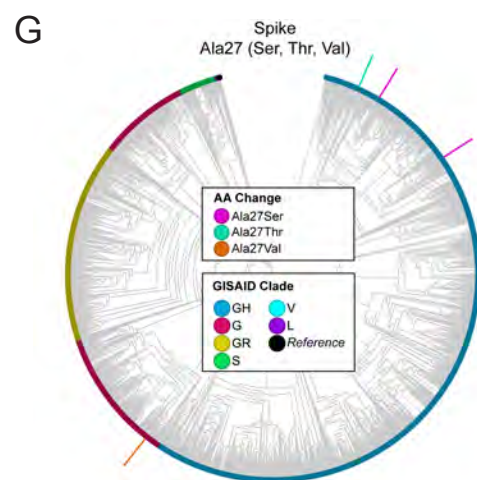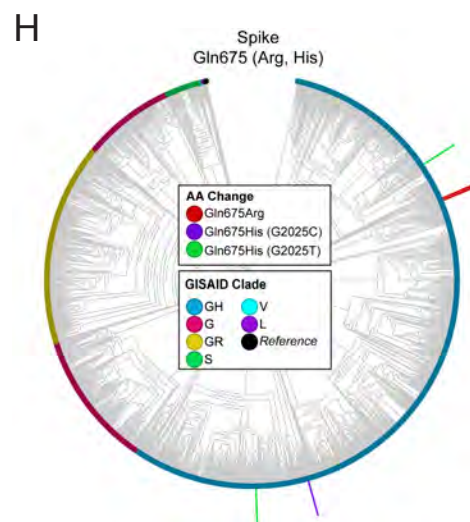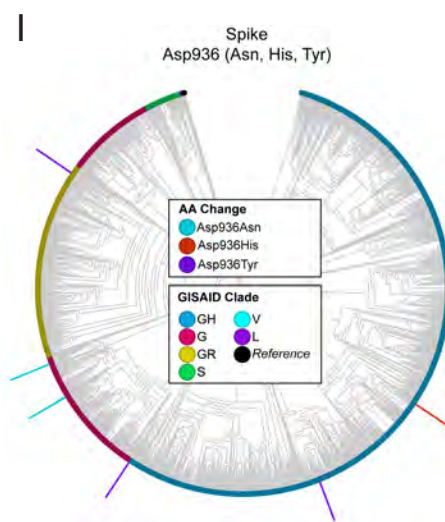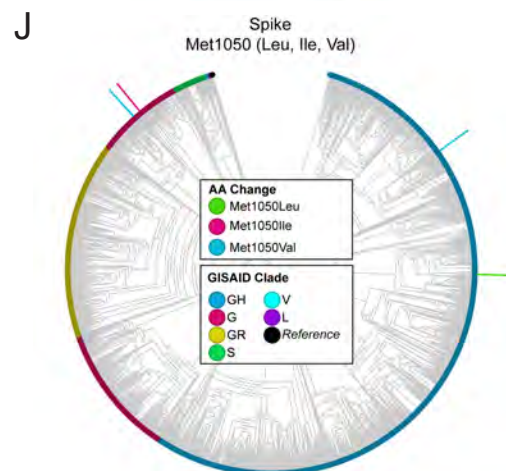

Supplement: FIG S5 [file mBio.02707-20-sf005.pdf]

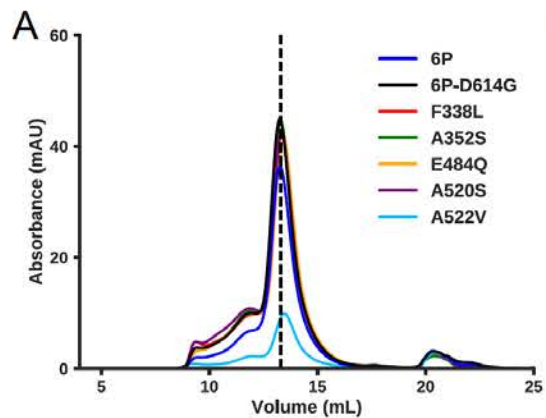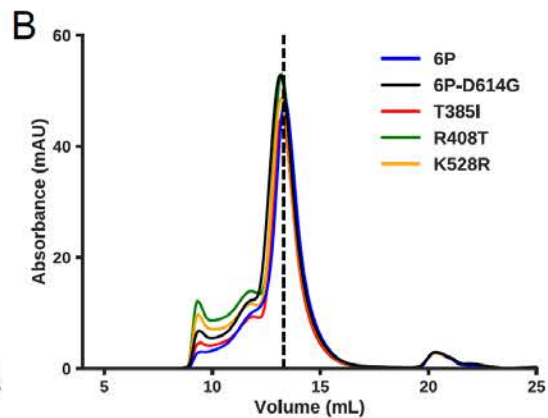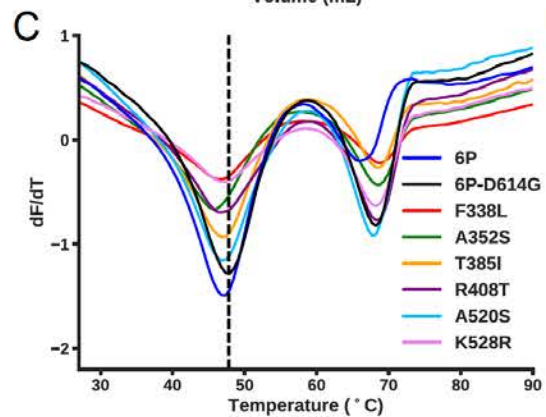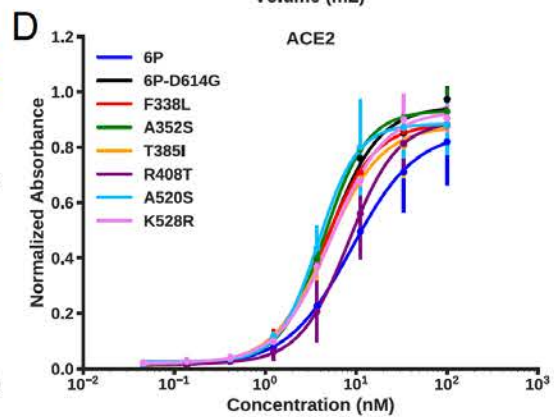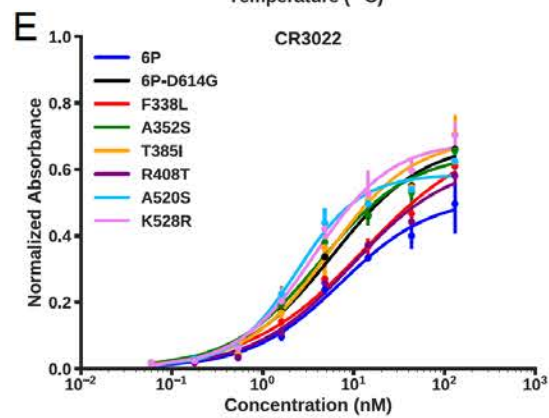

Supplement: FIG S6 [file mBio.02707-20-sf006.pdf]
