# Supplementary material for: Molecular Architecture of Early Dissemination and Massive Second Wave of the SARS-CoV-2 Virus in a Major Metropolitan Area
Source: mBio. 2020 Oct 30;11(6):e02707-20. doi: 10.1128/mBio.02707-20 (PMC7642679; doi:10.1128/mBio.02707-20)
Supplement: TABLE S2 [file mBio.02707-20-st002.pdf]

Supplemental Table 2. Classifier accuracy scores and performance information.

	Classification (F1 Scores)			Regression (R^2 Scores)		
	Patient Features	Genome Kmers	Both Features	Patient Features	Genome Kmers	Both Features
Model	Score	Score	Score	Score	Score	Score
Deceased[a]	0.53	0.45	0.55	-	-	-
Length of stay	-	-	-	-0.01	-0.16	-0.20
ICU[b]	0.60	0.44	0.54	-0.02	-0.08	-0.14
IMU[b]	0.505	0.41	0.42	-0.10	-0.10	-0.20
Ventilation[a]	0.55	0.46	0.55	-0.03	-0.11	-0.14
Oxygen[a]	0.53	0.49	0.50	-0.06	-0.05	-0.13
Wave[c]	0.54	0.57	0.57	-	-	-

[a]yes versus no

[b]classification models are based on admitted to unit versus never admitted to unit

[c]wave 1 versus wave 2
