# Supplementary material for: Molecular Architecture of Early Dissemination and Massive Second Wave of the SARS-CoV-2 Virus in a Major Metropolitan Area
Source: mBio. 2020 Oct 30;11(6):e02707-20. doi: 10.1128/mBio.02707-20 (PMC7642679; doi:10.1128/mBio.02707-20)
Supplement: TABLE S3 [file mBio.02707-20-st003.pdf]

Supplemental Table 3. Pearson Correlation Coefficients for Outcomes.

Category	Class	Deceased[a]	LengthOfStay[b]	IMU_LOS[b]	ICU_LOS[b]	MechVentilation[b]	SupplOxygen[b]
Sex	M	0.05	0.06	0.08	0.07	0.05	0.04
	F	-0.05	-0.06	-0.08	-0.07	-0.05	-0.04
ABO	A	-0.01	-0.02	0.02	-0.06	-0.05	-0.01
	B	-0.04	-0.04	-0.02	-0.03	-0.04	0.02
	AB	-0.01	0.00	-0.03	-0.03	0.04	-0.05
	O	0.05	0.05	0.00	0.09	0.06	0.02
Rh factor	Positive	0.03	0.00	-0.02	0.06	0.05	-0.03
	Negative	-0.03	0.00	0.02	-0.06	-0.05	0.03
Age	AGE	0.27	0.20	0.06	0.04	0.05	0.15
Ethnicity	Asian	0.01	-0.06	-0.02	-0.01	-0.01	-0.03
	Black	-0.03	-0.04	-0.03	-0.03	0.01	-0.10
	Caucasian	0.02	0.05	0.05	0.04	-0.01	0.08
	Hispanic	0.03	0.00	-0.01	0.02	-0.01	0.03
	Hawaiian/Pacific Islander	-0.02	0.07	0.01	0.00	0.02	0.12
	Native American	-0.01	0.01	-0.03	-0.03	-0.03	0.04
Ethnic Group	Hispanic or Latino	-0.01	0.00	0.00	0.06	0.00	0.07
	Not Hispanic or Latino	0.01	0.00	0.00	-0.06	0.00	-0.07
Wave	Wave1	0.08	0.18	-0.06	0.14	0.20	-0.13
	Wave2	-0.08	-0.18	0.06	-0.14	-0.20	0.13
A23403G	D614	0.01	0.06	-0.05	0.01	0.05	-0.06
	G614	-0.01	-0.06	0.05	-0.01	-0.05	0.06

[a]yes versus no
[b]days (LOS, length of stay)
