# Supplementary material for: Molecular Architecture of Early Dissemination and Massive Second Wave of the SARS-CoV-2 Virus in a Major Metropolitan Area
Source: mBio. 2020 Oct 30;11(6):e02707-20. doi: 10.1128/mBio.02707-20 (PMC7642679; doi:10.1128/mBio.02707-20)
Supplement: TABLE S4 [file mBio.02707-20-st004.pdf]

Supplemental Table 4A. Oligonucleotides used in this study.

Primer Name	Sequence
JK126_EcoRI_universal-F	aggcaacttcaagaacctgagagaattc
JK128_D614G-F-gib	Tgctgtaccaaggtgttaactgcac
JK129_D614G-R-gib	Gtgcagttaacaccttggtacagca
JK130_NheI_universal-R	Gtaggcgatgatgctctggctagc
JK131_A352S-F-gib	tgcctccgtttactcctggaatagaaag
JK132_A352S-R-gib	ctttctattccaggagtaaacggaggca
JK133_A419V-F-gib	cagaccggtaagatcgttgactacaactataa
JK134_A419V-R-gib	ttatagttgtagtcaacgatcttaccggtctg
JK135_A520S-F-gib	Ttactccactcccccgccac
JK136_A520S-R-gib	Gtggcgggggagtggagtaa
JK137_A522V-F-gib	Cacgcccccgtcaccgtctg
JK138_A522V-R-gib	Cagacggtgacgggggcgtg
JK139_E484Q-F-gib	Aacggggtgcaaggcttcaac
JK140_E484Q-R-gib	Gttgaagccttgcaccccgtt
JK141_F338L-F-gib	Ctctgccccctcggcgaggt
JK142_F338L-R-gib	Acctcgccgagggggcagag
JK143_F456L-F-gib	Taccggctgcttcgcaagtc
JK144_F456L-R-gib	Gacttgcgaagcagccggta
JK145_G446V-F-gib	Atagcaaggtggtcggcaactaca
JK146_G446V-R-gib	Tgtagttgccgaccaccttgctat
JK147_K528R-F-gib	Ctgcggtcctaggaagtccacc
JK148_K528R-R-gib	Ggtggacttcctaggaccgcag
JK149_R408T-F-gib	Cgatgaggtgacgcagatcgca
JK150_R408T-R-gib	Tgcgatctgcgtcacctcatcg
JK151_S373P-F-gib	Aacagcgcccctttcagcac
JK152_S373P-R-gib	Gtgctgaaaggggcgctgtt
JK153_T385I-F-gib	Ggcgtttccccaataaagctgaatga

JK154_T385I-R-gib	Tcattcagctttattggggaaacgcc
JK155_V445F-F-gib	Ggatagcaagttcggcggcaac
JK156_V445F-R-gib	Gttgccgccgaacttgctatcc

Supplemental Table 4B. Plasmids used in this study.

Plasmid Name	Spike Mutations	Reference
pIF637	F817P, A892P, A899P, A942P, K986P, V987P (or 6P)	PMC7402631
pIF638	6P, D614G	This study
pIF639	6P, D614G, F338L	This study
pIF640	6P, D614G, A352S	This study
pIF641	6P, D614G, T385I	This study
pIF642	6P, D614G, A419V	This study
pIF643	6P, D614G, V445F	This study
pIF644	6P, D614G, G446V	This study
pIF645	6P, D614G, F456L	This study
pIF646	6P, D614G, E484Q	This study
pIF647	6P, D614G, A520S	This study
pIF648	6P, D614G, A522V	This study
pIF649	6P, D614G, K528R	This study
pIF650	6P, D614G, S373P	This study
pIF651	6P, D614G, R408T	This study

Supplemental Table 4C. Summary of spike biophysical parameters.

	Tm*	ACE2 EC_{50}* (mean±SEM)	CR3022 EC_{50}* (mean±SEM)
6P	47.4	8.9 ± 1.9	7.3 ±1.9
6P, D614G	47.9	5.0 ± 0.4	5.9 ± 1.2
6P, D614G, F338L	46.8	4.5 ± 0.6	16 ± 6.6
6P, D614G, A352S	45,7	4.4 ± 0.3	4.3 ± 1.1
6P, D614G, T385I	46.8	4.8 ± 0.7	5.2 ± 1.4
6P, D614G, V445F	45.7	4.2 ± 0.7	5.4 ± 1.1
6P, D614G, G446V	44.5	4.0 ± 0.6	3.3 ± 0.5
6P, D614G, F456L	46.2	3.8 ± 0.2	4.0 ± 0.6
6P, D614G, E484Q	46.2	4.0 ± 0.5	4.0 ± 0.7
6P, D614G, A520S	46.8	3.8 ± 0.4	2.4 ± 0.3
6P, D614G, K528R	47.4	5.4 ± 0.4	3.6 ± 0.6
6P, D614G, S373P	46.8	3.8 ± 0.5	>70
6P, D614G, R408T	46.8	8.2 ± 1.3	10 ± 2.2

*Three biological replicates
